# N‑Heterocyclic Carbenes as Ligands to ^198^Au(I)-Radiolabeled Compounds: A New Platform for Radiopharmaceutical Design

**DOI:** 10.1021/acs.jmedchem.5c01073

**Published:** 2025-08-13

**Authors:** Sarah Spreckelmeyer, Sophie R. Thomas, Franziska Schuderer, Catarina I. G. Pinto, Ana Luiza de Andrade Querino, Felix A. Böhm, Mihyun Park, Christopher Geppert, Christian Gorges, Filipa Mendes, Angela Casini

**Affiliations:** † Department of Nuclear Medicine, Charité—Universitätsmedizin Berlin, Freie Universität Berlin, Humboldt-Universität zu Berlin, and Berlin Institute of Health, Augustenburger Platz 1, 13353 Berlin, Germany; ‡ Department of Inorganic Chemistry, University of Vienna, Währinger Straße. 42, 1090 Vienna, Austria; § Medicinal and Bioinorganic Chemistry, Department of Chemistry, School of Natural Sciences, 9184 Technical University of Munich, Lichtenbergstraße 4, 85748 Garching bei München, Germany; ∥ Pharmaceutical Radiochemistry, Department of Chemistry, School of Natural Sciences, Technical University of Munich, Walther-Meißner-Str. 3, 85748 Garching bei München, Germany; ⊥ C^2^TN—Centro de Ciências e Tecnologias Nucleares, Instituto Superior Técnico, 37809 Universidade de Lisboa, 2695-066 Lisboa, Portugal; # Forschungsreaktor TRIGA Mainz, 9182 Johannes Gutenberg-Universität Mainz, Fritz-Strassmann-Weg 2, 55128 Mainz, Germany; ¶ DECN—Departamento de Engenharia e Ciências Nucleares, Instituto Superior Técnico, Universidade de Lisboa, 2695-066 Lisboa, Portugal

## Abstract

The radionuclide ^198^Au, with a half-life of 2.7 days, emits γ radiation ideal for diagnostic purposes and generates β^–^ particles suitable for effective cancer radiotherapy, making it a perfect nuclide for “theranostics”. However, the application of coordination compounds of Au­(I)/Au­(III) in medicine is limited by their instability in vivo. Here, we explore N-heterocyclic carbene (NHC) organometallic chemistry to stabilize ^198^Au­(I) in radiopharmaceuticals. Thus, Au­(I) NHC compounds featuring different scaffolds were selected for ^198^Au radiolabeling. Eventually, two compounds featuring imidazole (**AuNHC-1**) and theophylline (**AuTMX**_**2**_) scaffolds were successfully radiolabeled (radiochemical purity = 92.9% and 40.2%, respectively). Instead, two peptidic Au­(I) benzimidazolylidene derivatives, capable of blood–brain barrier translocation in vitro, were subjected to ligand exchange reactions under the applied radiolabeling conditions. The obtained proof-of-concept results showed that NHCs are suitable ligands to achieve isotope exchange in Au­(I) complexes. Overall, our work reveals the still untapped potential of organometallic chemistry in radiopharmaceutical design.

## Graphical abstract

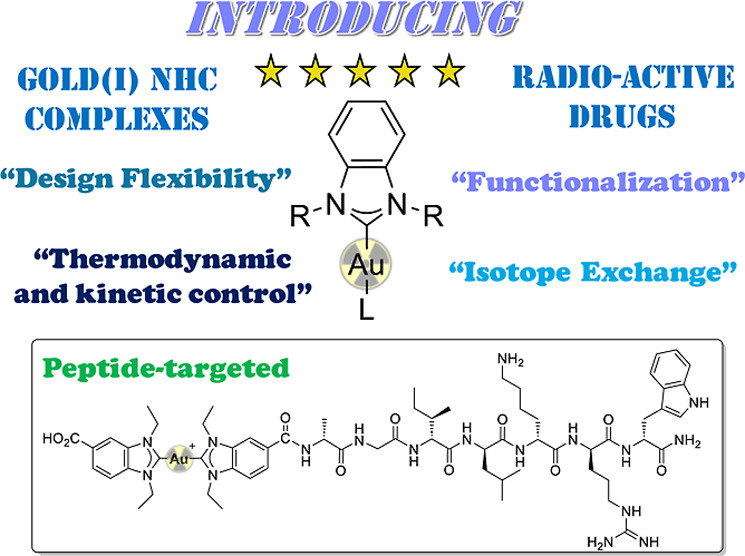

## Introduction

The position of Au in the Periodic Table of the Elements is unique owing to its large relativistic effects, increased ionization energies, and high redox potential, which set it apart from other neighboring elements.^1^ Particularly in the case of Au­(I) complexes, relativistic effects together with the lanthanide contraction (reducing the covalent radius of Au­(I)) strongly influence their geometric features (linear geometry), their electronic structure, and overall reactivity (“soft” Lewis acid). Accordingly, Au­(I) complexes bind preferentially to “soft” electron donor elements like P and S. Steric factors allowing Au­(I) compounds also have a marked tendency to interact with neighboring Au­(I) centers to give “*aurophilic*” interactions.^2^ On the other hand, for Au­(III), the relativistic effects are much less pronounced, and gold in this oxidation state displays all of the characteristics of a transition metal, adopting almost exclusively the square-planar coordination geometry common to other d^8^ ions, and its compounds are markedly different in terms of structure and reactivity from those of Au­(I).^3^ Due to such properties, the chemistry of gold has been one of the most striking research themes of recent times, leading to the development of gold-based molecules and materials for different uses.^4–8^

Among its noticeable properties, the radiochemistry of Au is also of potential interest for biomedical applications. Of the radioisotopes of Au, both ^198^Au and ^199^Au are medically useful because of their desirable nuclear properties.^9,10^ ^199^Au has a 3.14 day half-life and emits β^–^ particles (*E*_max_ = 452 keV) plus γ photons (*E*_γ_ = 158 keV, 72% and 208 keV, 22%), which are well suited to noninvasive single-photon emission computed tomography (SPECT) imaging.^11^ ^198^Au has a 2.7 day half-life and decays by emission of β^–^ particles (*E*_max_ = 1.37 MeV) and γ photons (*E*_γ_ = 412 keV, 98.9%). Although ^198^Au has been used for both SPECT and Cerenkov luminescence imaging, its abundant moderate-energy β^–^ emission and high-energy γ emission make it more suitable for therapeutic applications rather than for imaging. The production of carrier-added ^198^Au takes place in a reactor via the reaction of ^197^Au­(n,gamma)^198^Au, whereas ^199^Au can be obtained no-carrier added via ^198^Pt­(n,gamma)^199^Pt, (30 min half-life), which subsequently decays via beta emission to ^199^Au. For potential application in radionuclide therapy, a metal complex must be thermodynamically favored, as well as kinetically stable to ligand substitution and reductive decomposition. Unfortunately, the majority of Au­(I)/Au­(III) coordination compounds show limitations with respect to stability in physiological conditions in terms of kinetic lability, propensity to ligand exchange reactions with biological nucleophiles (e.g., water and protein thiolates),^12,13^ and redox stability. Both Au­(I) and Au­(III) coordination complexes can be reduced to Au(0) in a physiological environment, resulting in the release of both the free ligands and the metal, eventually leading to either a loss of activity or possible side effects.

In an attempt to circumvent such extensive *speciation* and to set the stage for practically useful Au-based bioactive compounds, the selection of the ligand framework of gold compounds is pivotal. However, to date, only one report by Jurisson and co-workers has described the application of radioactive ^198^Au­(III) molecular species using (bis)­thiosemicarbazone ligands.^14^ Nonetheless, these ligand systems were unsuitable for in vivo application due to the rapid degradation and reduction of Au­(III) to Au­(I) species.^14^ Previous studies with tetradentate Schiff base ligands were also shown to form ^198/199^Au­(III) complexes in 95–100% yield, although no further stability and imaging studies were conducted.^15^ In 2014, a study involving graphene oxide radiolabeled with a mixture of ^199^Au­(III) and ^198^Au­(III) ions tethered to graphene via binding to amino groups of a 3-aminopropyl-trimethoxysilane linker showed potential for SPECT tumor imaging.^16^ Concerning radioactive Au­(I) complexes, early studies report on water-soluble phosphine ligands, which were evaluated to form Au­(I) complexes with a tetrahedral geometry.^17^ However, biodistribution studies were performed in rats and showed extensive decomposition due to ligand exchange reactions with intracellular thiols.^18^ In another study, a dinuclear bisphosphino Au­(I) dithiocarbamate was successfully radiolabeled with ^198^Au­(I).^19^ Unfortunately, this compound showed unfavorable biodistribution in the lungs, liver, and spleen in rats. In a recent study by Baitullina et al., coordination polyhedra with the motif 2,6-dipicolinoyl bis­(*N*,*N*-dialkylthioureas) were investigated among others toward their radiolabeling abilities with ^68^Ga, ^177^Lu, and ^198^Au­(I).^20^ As a result, radiolabeling experiments were successful, but low stability in human serum albumin was observed, hindering their further application.

To avoid metal ion complexation, gold nanoparticles (AuNPs) have also been used to incorporate ^198^Au, enabling the quantification of their in vivo biodistribution and tumor uptake in mouse models by measuring the γ radiation from ^198^Au decay and, in some cases, by optical imaging through the detection of the Cerenkov radiation.^21–24^ Interestingly, receptor-targeted ^198^AuNPs have shown important tumor reduction in xenograft models of prostate cancer.^25^ In 2016, the first synthesis of AuNPs doped with ^199^Au atoms for targeted SPECT tumor imaging in a mouse triple-negative breast cancer model was reported.^26^ Despite these encouraging results, the use of AuNPs for radionuclide therapy has some drawbacks, including challenging administration routes, possible toxicity due to the accumulation of colloids in off-target organs, and the inherent difficulties in analyzing nanoscale materials.^27,28^ For example, the necessity to stabilize the AuNPs and make them targeted toward the tumor site via the judicious choice of the coating strategy requires extensive characterization of the nanomaterials via different analytical and spectroscopic methods.

To further develop nuclear medicine applications of Au­(I) complexes, we envisaged that the use of organometallic chemistry to stabilize the gold radioisotope via the formation of a direct metal–carbon bond would be advantageous. Therefore, synthesizing a family of radioactive organometallic ^198^Au­(I) N-heterocyclic carbene (NHC) complexes was attempted starting from their cold analogs. It is worth mentioning that NHCs have emerged as ideal ligands not only thanks to their remarkable σ-donor and π-back-donation ability,^29^ necessary to stabilize Au­(I) ions in physiological conditions, but also since they perfectly fit the prerequisites for an efficient (radio)­pharmaceutical design enabling modulation of the metal compound’s reactivity and physicochemical properties.^30–37^ To fine-tune the strength of the carbene–metal bond and, therefore, the stability of the NHC-metal fragment in aqueous solution, derivatization of the backbone scaffold has a central influence, whereas modifications of the wingtip positions can change the steric properties of the complex to protect the carbene-metal bond and enhance the stability (Figure 1A). Furthermore, by varying the side chains of the wingtip groups as well as of the backbone positions, it is possible to influence the NHC lipophilicity as well as its functionalization with bioactive moieties relevant to (radio)­pharmaceutical design (e.g., fluorophores for microscopy and peptides/antibodies for targeting).^38–40^ Beyond this versatility, the ease of synthesis of Au­(I) NHC complexes, combined with their inherent stability, renders this family of organometallic compounds very appealing.

**Figure 1:**
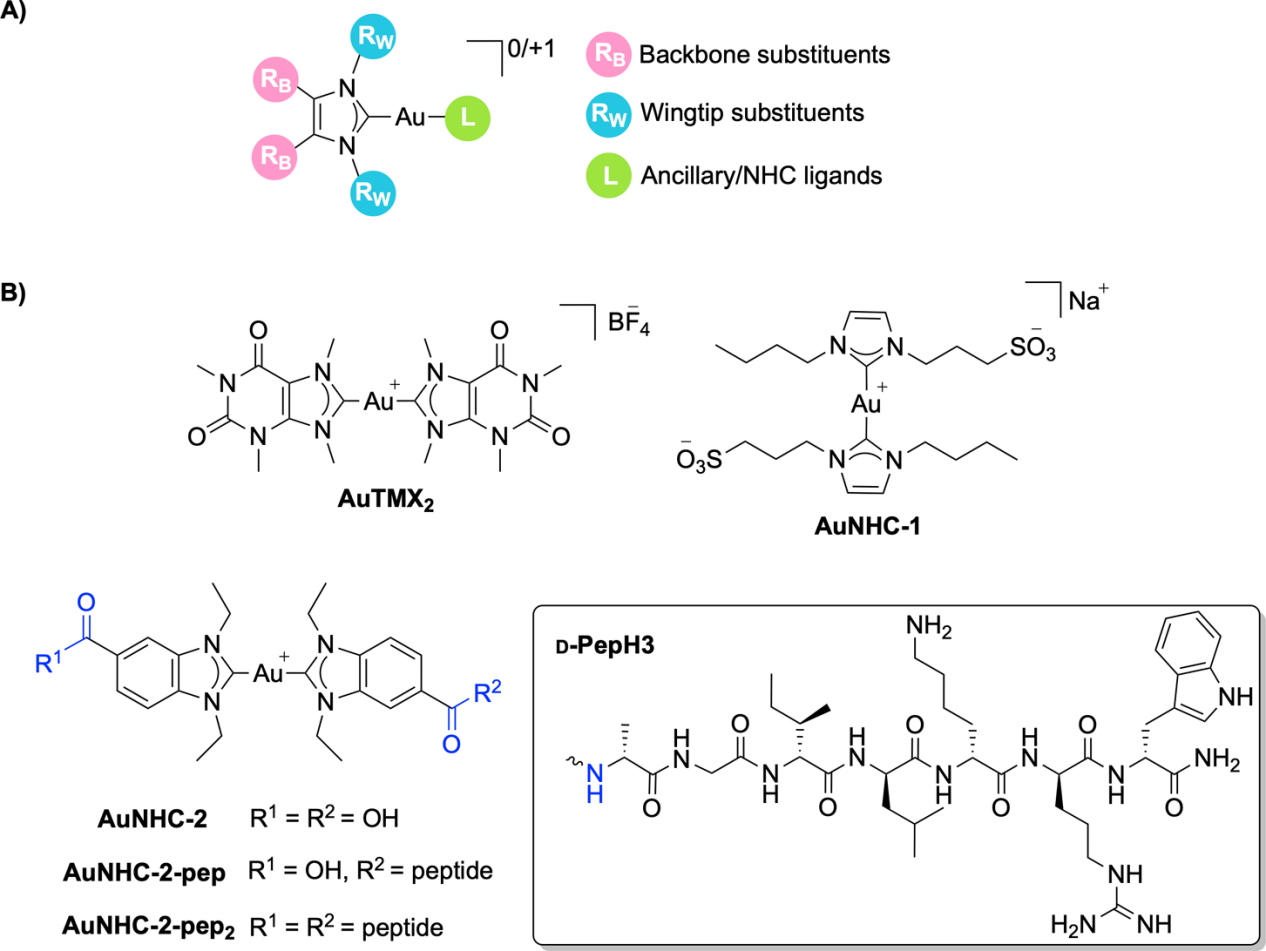
(A) General structure of Au­(I) NHC complexes with possible sites for derivatization highlighted. (B) Structures of the Au­(I) NHC compounds investigated in this study: **AuTMX**_**2**_, **AuNHC-1**, **AuNHC-2**, and bioconjugated compounds **AuNHC-2-pep** and **AuNHC-2-pep**_**2**_ (pep = d-PepH3-NH_2_).

Here, we have selected organometallic bis-NHC Au­(I) complexes representative of different families: namely, **AuTMX**_**2**_ ([Au­(9-methylcaffeine-8-ylidene)_2_]^+^), a cytotoxic complex capable of selectively stabilizing telomeric DNA G-quadruplex structures,^41^ and **AuNHC-1**, a water-soluble complex with a sulfonate group on one of the wingtips^42^ (Figure 1B). Moreover, a novel bis-carbenic complex with carboxylic acid groups on the backbone to allow for bioconjugation (**AuNHC-2**, Figure 1B, Scheme 1A) was also synthesized. In a proof-of-concept experiment to demonstrate the potential of Au­(I) NHC complexes to develop peptide-targeted radiopharmaceuticals,^43,44^ **AuNHC-2** was conjugated to a peptide able to cross the blood–brain barrier (BBB), **d** **-PepH3-NH**_**2**_ (H_2_N-AGILKRW-NH_2_), forming **AuNHC-2-pep** and **AuNHC-2-pep**_**2**_ (Figure 1B, Scheme 1B).^45^ The use of peptidic vectors to guide radiopharmaceuticals to the brain is attractive for the imaging and therapy of brain cancers. In this context, PepH3 has previously been studied for its BBB translocation capability in vitro and in vivo.^45,46^ Interestingly, the peptide was shown to cross the BBB via a receptor-independent mechanism, namely, via adsorptive-mediated transcytosis,^47^ due to the presence of positively charged amino acid residues in its sequence. The two bioconjugated Au­(I) complexes were studied for their BBB-translocation capabilities using an in vitro model. Further, a protocol to achieve [^198^Au]­AuNHC complexes was optimized for compounds **AuTMX**_**2**_, **AuNHC-1**, and **AuNHC-2**, as well as the targeted **AuNHC-2-pep**. For our preliminary radiotracer studies, carrier-added ^198^Au was used rather than the high-specific-activity ^199^Au due to easier production and reduced cost.

**Scheme 1:**
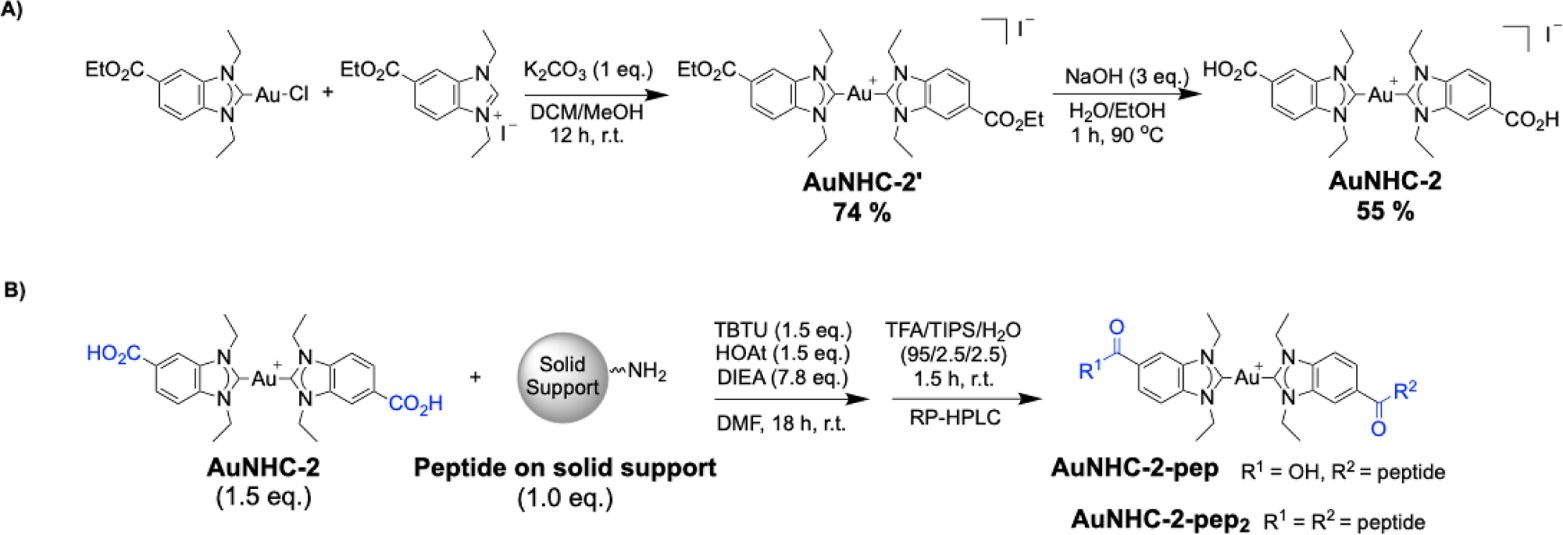
(A) Synthesis Scheme of Compound**AuNHC-2**; (B) synthesis of the Bioconjugated**AuNHC-2-pep** and **AuNHC-2-pep**_**2**_ by Amide Bond Formation on Solid Support between **AuNHC-2** and **d** **-PepH3-NH**_**2**_, Followed by Cleavage from the Resin

## Results

### Synthesis and Characterization of Cold Au­(I) NHC Complexes

Initially, the Au­(I) NHC compounds were synthesized according to established (**AuTMX**_**2**_ and **AuNHC-1**)^41,42^ or adapted (**AuNHC-2**, Scheme 1A)^48^ procedures and characterized by standard methods (see the Experimental Section for details, Figures S1–S3). Moreover, they were purified by high-performance liquid chromatography (HPLC), and UV chromatograms were recorded (Figures S4–S6). For the synthesis of **AuNHC-2-pep**, a typical amide coupling reaction between **AuNHC-2** and **d** **-PepH3-NH**_**2**_ was performed on the resin (solid support) to reduce the number of side reactions (Scheme 1B). After cleavage from the resin, the crude reaction mixture was purified by reverse-phase HPLC (RP-HPLC) to obtain both **AuNHC-2-pep** (5.6%, Figures S7–S9) and **AuNHC-2-pep**_**2**_ (6.8%, Figures S10–S14).

### Evaluation of Au­(I) Complexes in an In Vitro BBB Cell Model

Further, the evaluation of the ability of the two bioconjugated Au­(I) complexes to translocate the BBB was performed in the bEnd.3 cell model in vitro.^49^ We selected the brain endothelioma bEnd.3 murine cell line as a representative established cell line due to its facile growth and ability to maintain BBB characteristics and functional barriers. As described previously by us, this in vitro BBB model was established by growing the bEnd.3 cells in fibronectin-coated transwell filters enabling the growth of a tight monolayer of cells (Figure 2A).^50^ Before exploring BBB translocation, a cell viability assay was performed to assess the toxicity of the Au­(I) complexes in this cell line grown under normal culture conditions. No cytotoxicity was observed after 24 h of incubation with compound concentrations of 1–25 μM (Figure S15). To assess the ability of the complexes to translocate the in vitro BBB model, bEnd.3 cells were exposed to different concentrations of **AuNHC-2-pep** and **AuNHC-2-pep**_**2**_ complexes for 30 min and 2 h, and the Au content in the apical and the basolateral media was determined by inductively coupled plasma mass spectrometry (ICP–MS). The translocation efficiency was determined by the amount of Au detected in the basolateral medium as a % of the total Au content recovered (apical + basolateral media) (Figure 2B).

**Figure 2:**
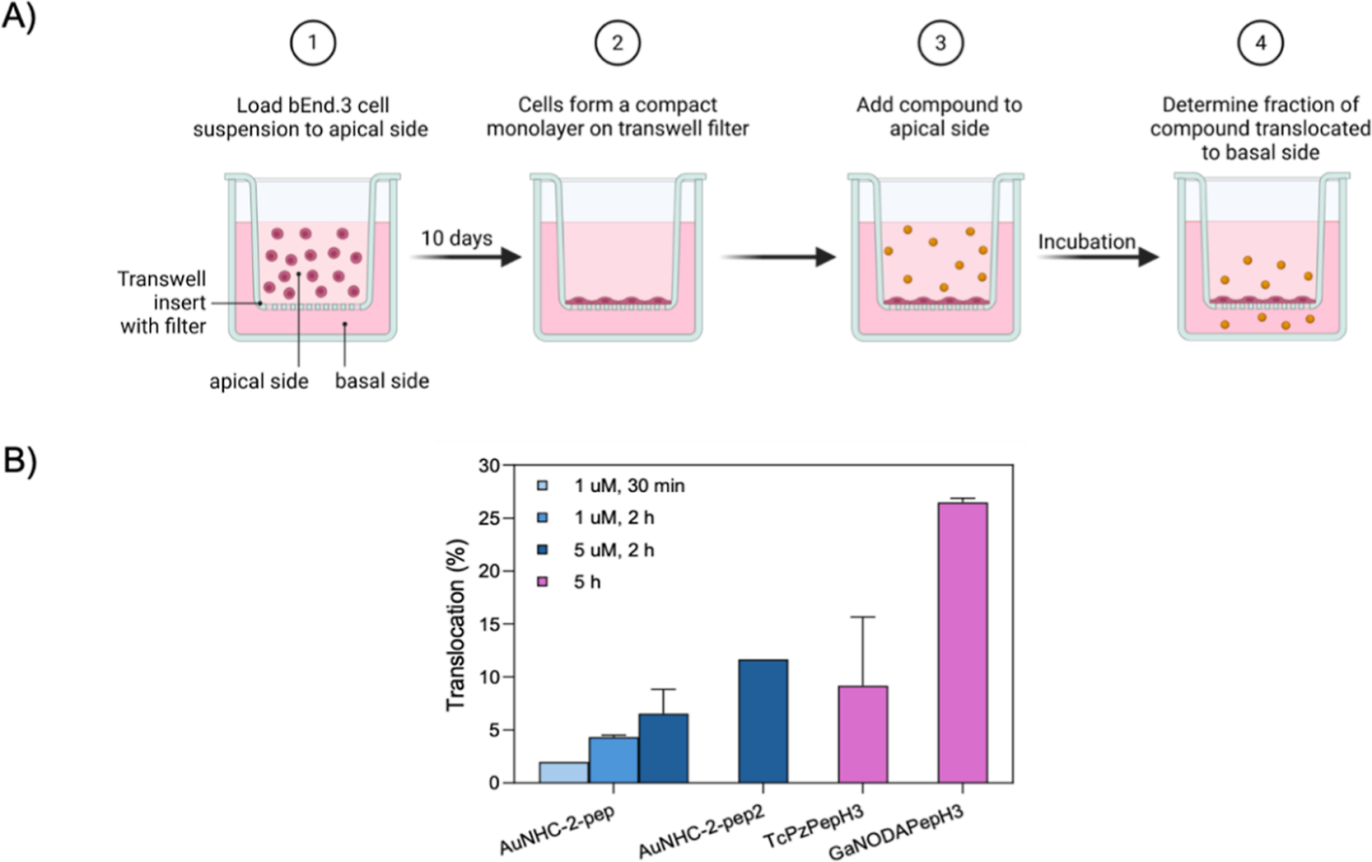
(A) Scheme of the in vitro BBB cellular model translocation assay (created in BioRender). (B) Translocation of Au complexes in the in vitro BBB model, expressed as % of the total compound recovered. Values for TcPzPepH3 and GaNODAPepH3 are from ref 46.

Overall, the ICP–MS determination of the Au content showed that both complexes can translocate the in vitro BBB cell model, although to a different extent, with **AuNHC-2-pep**_**2**_ presenting a higher translocation value. The amount of Au recovered in the apical and basolateral media was always >98% of the total applied, which suggests that no Au (and therefore, no Au­(I) complex) is retained intracellularly, nor in the insert. Additionally, a post-translocation integrity assay was performed to evaluate the integrity of the endothelial barrier by assessing the permeability of fluorescently labeled dextran (FD) with a 4 kDa molecular weight (FD4). No decrease in the integrity of the cell model (>80%) was detected, ensuring that the Au­(I) complexes were not promoting any disruption of the BBB in the in vitro model. These data are in line with previous studies whereby PepH3 bioconjugate tracers of ^99m^Tc and ^67^Ga showed BBB translocation values of ∼10–26% after 5 h of incubation (Figure 2B).^46^

### ^198^Au-Radiolabeling of Au­(I) NHC Complexes and Stability Studies

Finally, we attempted the radiolabeling of selected Au­(I) NHC compounds according to the experimental protocol depicted in Figure 3A,B. The results of ^198^Au-radiolabeling are shown in Figure 4, where the radio-HPLC chromatograms are presented. The starting material [^198^Au]­Au­(THT)Cl (THT = tetrahydrothiophene) has a retention time of 7.1 min (Figure 4A), whereas the radio-chromatogram of complex [^198^Au]**AuTMX**_**2**_ (Figure 4B) shows the product peak at the expected retention time of t_R_ = 8.0 min, matching the UV signal of the nonradioactive precursor (Figure S4). However, the peak of the starting material [^198^Au]­Au­(THT)Cl is still present, leading to a radiochemical purity (RCP) of only 40.2%. Compound [^198^Au]**AuNHC-1** (Figure 4C) shows a clear peak at *t*_R_ = 8.1 min, in agreement with the retention time of nonradioactive **AuNHC-1** in the UV chromatogram (*t*_R_ = 7.7 min, Figure S5). The discrepancy between *t*_R_ = 8.1 min and *t*_R_ = 7.7 min is likely due to the experimental setup of the HPLC system, whereby the injected solution passes first through the UV detector followed by the radioactivity detector. The resulting RCP is 92.9%, as assessed by radio-HPLC. Further, the stability of [^198^Au]**AuNHC-1** was assessed in the presence of human serum albumin (Figure 3C): ca. 57% of the compound remained stable after 1 h of incubation. With radio-HPLC, we confirmed the presence of our intact ^198^Au-radiolabeled complex (*t*_R_ ca. 8 min) and a minor new peak at *t*_R_ = 9.8 min (Figure S16).

**Figure 3:**
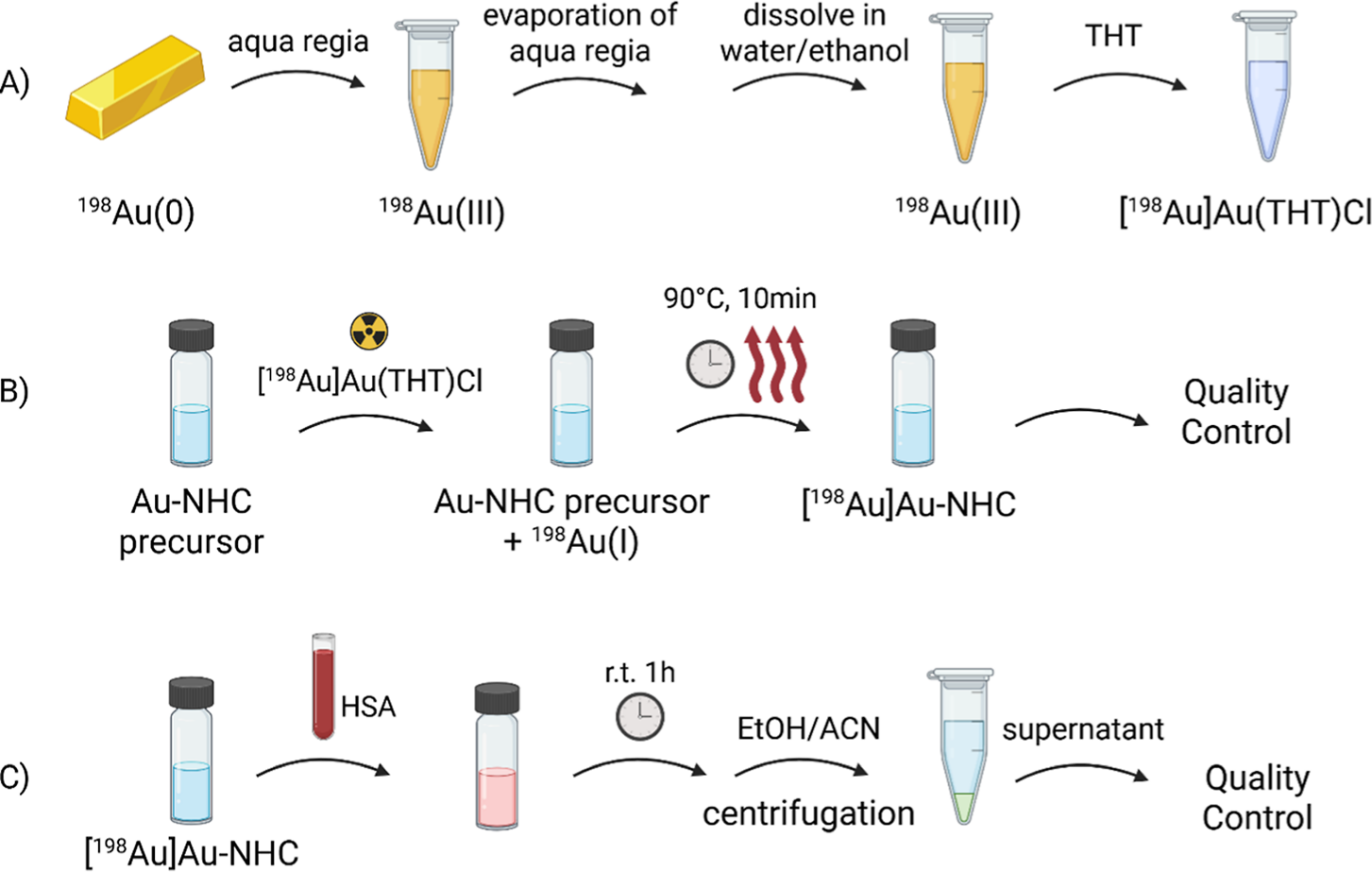
Schematic experimental setup of (A) preparation of [^198^Au]­Au­(THT)Cl and (B) ^198^Au-radiolabeling of Au­(I) NHC precursors. (C) Setup of the stability experiment with human serum albumin (HSA). Quality control performed by radio-HPLC (created in BioRender).

**Figure 4:**
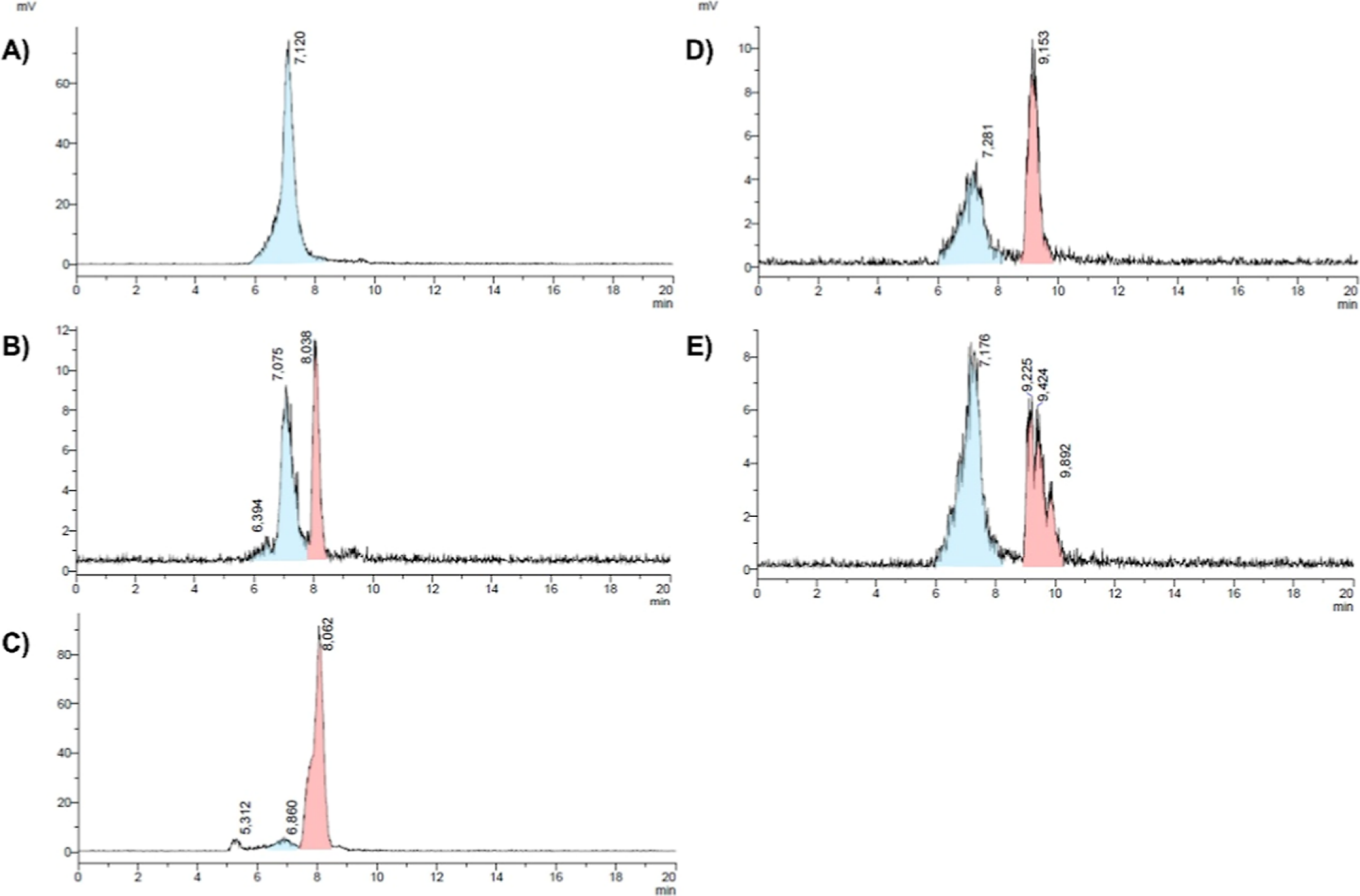
Radio-HPLC chromatogram of (A) [^198^Au]­Au­(THT)­Cl (blue trace), (B) [^198^Au]**AuTMX**_**2**_, (C) [^198^Au]**AuNHC-1**, (D) [^198^Au]**AuNHC-2**, and (E) [^198^Au]**AuNHC-2-pep** (red trace).

Unfortunately, the radiolabeling of **AuNHC-2** and **AuNHC-2-pep** did not lead to the desired radiolabeled products [^198^Au]**AuNHC-2** and [^198^Au]**AuNHC-2-pep**. In fact, while the retention time of the nonradioactive complexes are *t*_R_ = 10.8 and 10.0 min for **AuNHC-2** (Figure S6) and **AuNHC-2-pep** (Figure S7), respectively, the radio-chromatograms (Figure 4D,E) showed radioactive species at *t*_R_ = ca. 9.2 min. Moreover, in both cases, the presence of [^198^Au]­Au­(THT)­Cl is still detected.

In order to elucidate the nature of the observed possible byproducts, we conducted further stability experiments by HPLC electrospray ionization mass spectrometry (HPLC-ESI-MS) on the two nonradioactive complexes working in radiolabeling conditions (90 °C for 10 min in dimethyl sulfoxide (DMSO)/water/EtOH). In summary, when **AuNHC-2** and **AuNHC-2-pep** were heated to 90 °C for 10 min in DMSO, the compounds remained stable (middle traces in Figures 5A,B and 6A,B, *t*_R_ = 9.4 min for **AuNHC-2** and *t*_R_ = 8.8 min for **AuNHC-2-pep**). However, when the [Au­(THT)­Cl] solution was added and heated to 90 °C for 10 min in DMSO, both compounds showed the formation of different species due to ligand exchange reactions of the parent Au­(I) NHC compound. Specifically, we could observe the formation of [Au­(THT)­(MeCN)]^+^ and monocarbene [Au­(NHC)­(MeCN)]^+^ adducts for both Au­(I) NHC complexes (Figure 5: *t*_R_ = 5.6 and 7.7 min, respectively, for **AuNHC-2** and Figure 6: *t*_R_ = 5.7 and 7.8 min, respectively, for **AuNHC-2-pep**). Furthermore, both complexes exhibited a peak at 9.3/9.4 min, corresponding to low molecular weight masses, most likely due to adducts between DMSO and Au­(THT)­Cl. Finally, **AuNHC-2-pep** showed an additional species at 8.0 min (Figure 6), corresponding to the positively charged gold complex with retainment of the monocarbene peptide ligand.

**Figure 5:**
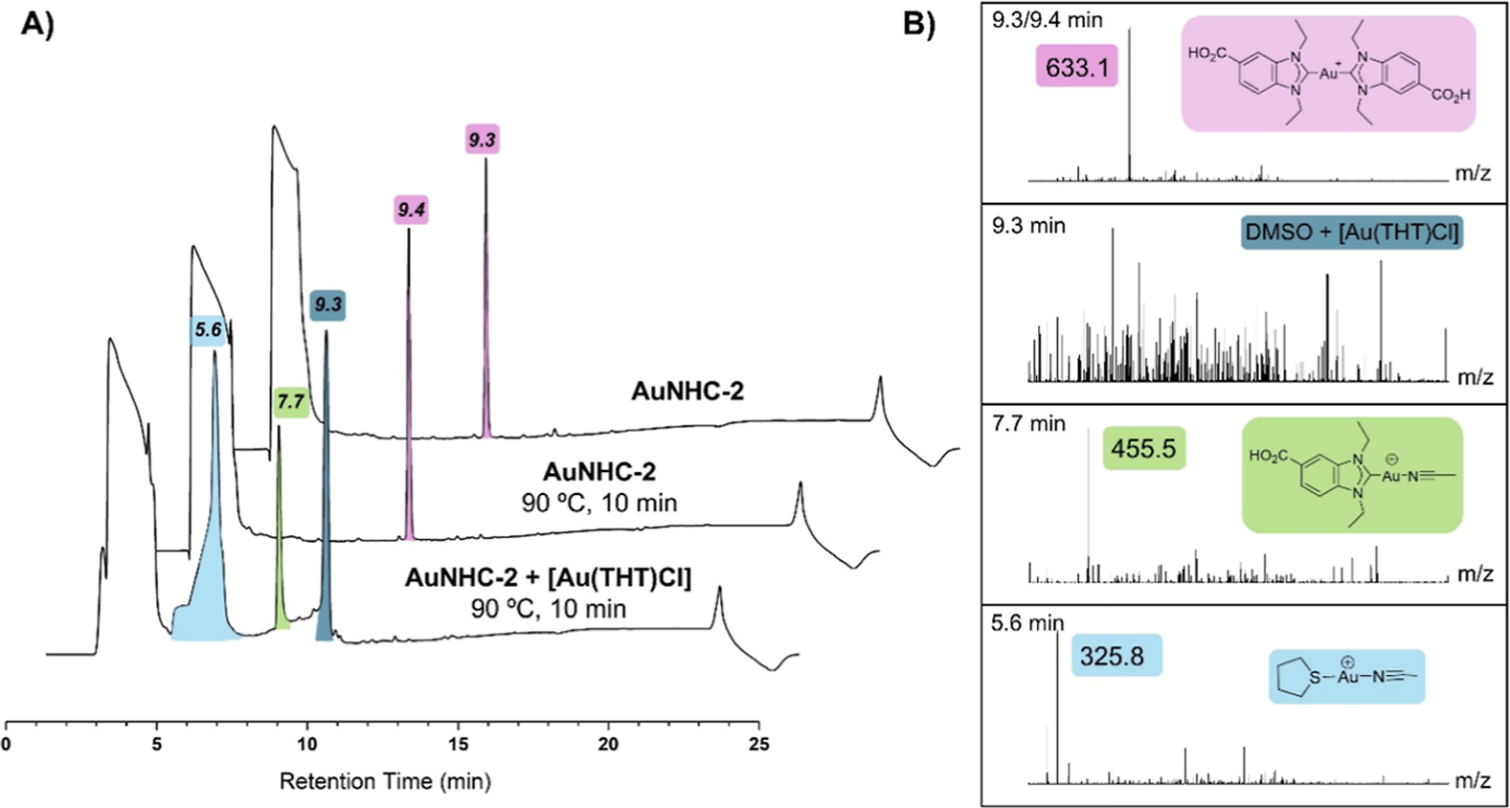
Stability of compound **AuNHC-2** in different conditions studied by HPLC-ESI-MS. (A) Stack overlay of RP-HPLC chromatograms of **AuNHC-2** with different treatment conditions. The runs were performed with water (0.1 vol % trifluoroacetic acid (TFA)) and MeCN (0.1 vol % TFA) at a gradient of 0–100 over 15 min. Peaks with their corresponding retention times are color-coded depending on the species formed. (B) Stacked ESI-MS spectra for isolated peaks from RP-HPLC chromatograms with main species highlighted with the corresponding structure.

**Figure 6:**
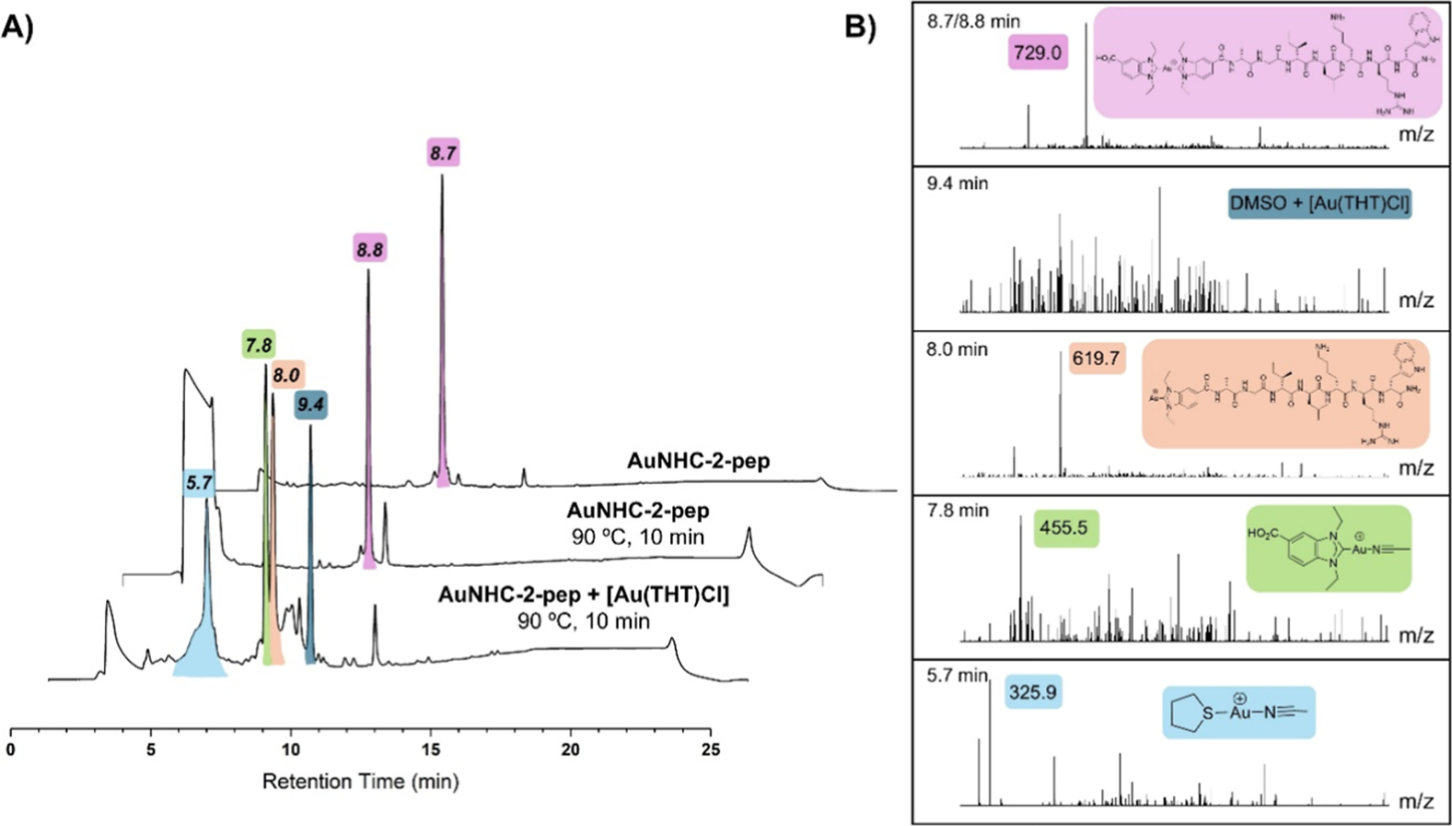
Stability of compound **AuNHC-2-pep** in different conditions studied by HPLC-ESI-MS. (A) Stack overlay of RP-HPLC chromatograms of **AuNHC-2-pep** with different treatment conditions. The runs were performed with water (0.1 vol % TFA) and MeCN (0.1 vol % TFA) at a gradient of 0–100 over 15 min. Peaks with their corresponding retention times are color-coded depending on the species formed. (B) Stacked ESI-MS spectra for isolated peaks from RP-HPLC chromatograms with main species highlighted with the corresponding structure.

Overall, our studies show that the efficiency of the radiolabeling protocol is highly susceptible to the type of the NHC scaffold, e.g., imidazolium- vs benzimidazole-based NHC, type of wingtip groups, and overall steric hindrance at the carbenic center. The nature of the NHC affects both the efficiency of the isotope exchange process and the stability of the resulting radioactive species. Further studies are necessary to obtain structure–activity relationships and fully unravel the determinants in the obtainment of ^198^Au­(I) NHC complexes. Moreover, optimization of the radiolabeling protocol could be envisaged to avoid the observed speciation effects for specific compounds. For example, the generation of ^198^Au­(I) precursors using less nucleophilic ligands compared to THT could be explored.

## Conclusions

Due to the lack of stability of previously reported radioactive Au-coordination complexes, there are currently no ^198/199^Au-radiopharmaceuticals in clinical use. In our study, we selected five organometallic Au­(I) compounds based on different NHC ligands, endowed with higher stability with respect to classical coordination complexes. In order to showcase the robustness of the NHC scaffold with respect to functionalization, essential to achieve targeted radiopharmaceuticals, two Au­(I) complexes with benzimidazole-type NHCs were obtained, featuring a short peptide sequence able to cross the BBB. The BBB translocation capability of the two bioconjugated Au­(I) compounds was ascertained in vitro.

Of note, we achieved the ^198^Au-radiolabeling of two Au­(I) NHCs complexes in our selection based on imidazole (**AuNHC-1**) and theophylline (**AuTMX**_**2**_) scaffolds. Formation of radiolabeled products was also accomplished in the case of benzimidazole-derived Au­(I) NHCs; however, in this case, extensive ligand exchange reactions prevented the obtainment of the target radiolabeled compounds. Stability studies on the “cold” Au­(I) NHC compounds, conducted in the same conditions as the radiolabeling experiments, enabled the identification of the different species formed in solution and allowed us to attribute the observed speciation to the addition of the highly nucleophilic [Au­(I)­(THT)­Cl] precursor.

The obtained proof-of-concept studies showed that NHCs are suitable ligands to achieve isotope exchange in Au­(I) complexes, with imidazole derivatives being most effectively radiolabeled and stable, also in the presence of Au­(THT)Cl and human serum albumin. Although further studies are necessary to optimize the NHC ligand design to improve stability in different conditions, our preliminary results, together with the great variety of NHC ligands available in the literature, give reasonable assumptions that Au­(I) NHCs might be a game-changer regarding access to radioactive gold compounds for radiopharmaceutical applications. Radiolabeling of cytotoxic gold compounds may also be relevant to achieve biodistribution studies in vivo to elucidate their mode of actions and therapeutic effects in a variety of diseases.^51^ Moreover, NHC ligands can also be envisaged to stabilize Au­(III) ions,^52^ further broadening the scope of radioactive gold compounds’ design and applicability.

## Experimental Section

### General Procedure

Solvents and reagents (reagent grade) were all commercially available and used without further purification. Reactions using dry solvents were performed under nitrogen in flame-dried glassware. Reactions involving silver or gold were carried out in the absence of light. Flash column chromatography was performed on silica gel 60A (particle size, 40–63 μm). Additionally, thin-layer chromatography was performed using Merck Millipore silica gel 60 F-254 plates and analyzed with UV light. ^1^H and ^13^C­{^1^H} NMR spectra were recorded on a Bruker AVANCE DPX 400 at ambient temperature. Chemical shifts δ are reported in parts per million (ppm), and coupling constants *J* are reported in Hertz (Hz), with residual ^1^H and ^13^C signals corresponding to the deuterated solvents as internal standards. Elemental Analysis (EA) for C, H, and N was performed by the microanalytical laboratory at the Technical University of Munich. HR-ESI-MS was carried out on a Thermo Fisher Exactive Orbitrap mass spectrometer equipped with a Thermo Fisher ESI source. Samples were prepared in acetonitrile or a mixture of acetonitrile/H_2_O (1:1) and syringe-filtered before direct injection; ions were detected in the positive mode. HR-Desorption Electrospray Ionization (DESI)-MS experiments were performed on a Q Exactive Plus mass spectrometer (Thermo Fisher Scientific Inc., Bremen, Germany) operated in the positive ion mode. Data was obtained with a resolution of 70,000 within the mass-to-charge (*m*/*z*) range of 600–1000. The capillary temperature was set to 320 °C, the S-Lens RF value to 100, and the maximum injection time to 250 ms. A mixture of HPLC grade methanol and water (95:5, v/v) was used as the electrospray solvent at a flow rate of 1.50 μL/min and a spray voltage of 4.5 kV. The nebulizing gas pressure was set to 7 bar (Nitrogen N5.0). The DESI geometrical parameters are as follows: a sprayer-to-sample distance of 1.5 mm, a sprayer-to-inlet distance of 6 mm, a spray angle of 75°, and a collection angle of 10°.

The Au­(I) NHC complexes (**AuTMX**_**2**_, **AuNHC-1**, and (5-(ethoxycarbonyl)-1,3-diethyl-2,3-dihydro-1*H*-benzo­[*d*]­imidazole-2-yl)­gold­(I) chloride) and the peptide, **d** **-PepH3-NH**_**2**_ (H_2_N-aGilkrw-NH_2_), were synthesized following the established protocols,^41,42,45,46,53^ and their purity was assessed to be >97% (by EA or HPLC-HR-ESI-MS).

### Synthesis and Characterization

#### Synthesis of **AuNHC-2**

**AuNHC-2** was synthesized following an adapted procedure published by Ott and co-workers (Scheme 1A).^48^ In detail, (5-(ethoxycarbonyl)-1,3-diethyl-2,3-dihydro-1*H*-benzo­[*d*]­imidazole-2-yl)­gold­(I) chloride (118.5 mg, 0.25 mmol) and its corresponding ligand (93.6 mg, 0.25 mmol) were stirred with K_2_CO_3_ (34.6 mg, 0.25 mmol) in a DCM/MeOH (1:1, 6 mL) mixture at room temperature for 12 h. The mixture was subsequently filtered before the product was isolated by using column chromatography (DCM/MeOH 9:1). Yield: 125 mg (0.15 mmol, 74%) of orange powder. The deprotection of **AuNHC-2** was then performed using a modified procedure published by Crudden and co-workers (Scheme 1A).^54^ **AuNHC-2** (28 mg, 0.034 mmol) and NaOH (5.5 mg, 0.138 mmol) were heated to 90 °C for 1 h in EtOH/H_2_O (1:1, 10 mL). After cooling, EtOH was removed under reduced pressure before acidification with 1 M HCl to obtain pH 1–2. The aqueous solvent was subsequently removed under reduced pressure, and the resulting solid was washed with methanol until the filtrate became colorless. Yield: 14 mg (0.018 mmol, 55%) white powder. Purity >95%.

^1^H NMR (400 MHz, DMF-*d*_7_): δ 8.59 (s, 2H, Ar), 8.23 (dd, *J* = 8.6, 1.4 Hz, 2H, Ar), 8.15 (d, *J* = 8.6 Hz, 2H, Ar), 4.90 (dq, *J* = 22.6, 7.3 Hz, 8H, NC*H*_2_CH_3_), 1.70 (q, *J* = 6.9 Hz, 12H, NCH_2_C*H*_3_).

^13^C NMR (101 MHz, DMF-*d*_7_): δ 193.44 (2C, N*C*N), 168.03 (2C, *C*OO), 136.87 (2C, *C*(Ar)), 134.05 (2C, *C*(Ar)), 129.09 (2C, *C*(Ar)), 127.07 (2C, *C*(Ar)­H), 114.99 (2C, *C*(Ar)­H), 113.39 (2C, *C*(Ar)­H), 45.11 (2C, N*C*H_2_CH_3_), 45.00 (2C, N*C*H_2_CH_3_), 16.98 (2C, NCH_2_ *C*H_3_), 16.78 (2C, NCH_2_ *C*H_3_).

HR-ESI-MS (CH_3_CN, pos. mode): C_24_H_28_AuN_4_O_4_^+^ (**AuNHC-2**) [M+]: *m*/*z* = exp 633.1749 (calcd 633.1775).

EA: Calcd for C_24_H_30_AuIN_4_O_5_ (**AuNHC-2** + 2H_2_O) [%] C, 36.20; H, 4.05; N, 7.04. Found [%]: C, 36.10; H, 3.89; N, 6.93.

#### Synthesis of **AuNHC-2-pep** and **AuNHC-2-pep**_**2**_

**AuNHC-2** (1.5 equiv, 0.02 mmol) was dissolved in 4 mL of dimethylformamide together with 2-(1*H*-Benzotriazole-1-yl)-1,1,3,3-tetramethylaminium tetrafluoroborate (TBTU, 1.5 equiv, 0.02 mmol) and 1-Hydroxy-7-azabenzotriazole (HOAt, 1.5 equiv, 0.02 mmol), and the pH was adjusted to pH = 9 with *N*,*N*-diisopropylethylamine (DIEA, 7.8 equiv, 0.10 mmol) (Scheme 1B). The coupling to the *n* -terminus of **d** **-PepH3-NH**_**2**_ (H_2_N-aGilkrw-NH_2_) via manual solid-phase peptide synthesis was performed over 18 h at r.t. After cleavage off the resin using 10 mL of TFA/Triisopropyl silane/H_2_O (95/2.5/2.5) for 1.5 h (30 min fraction 1 + 1 h fraction 2; 5 mL each) and evaporation of the solvent under nitrogen current, the crude product was purified using RP-HPLC (30–70% MeCN (+5% H_2_O, + 0.1% TFA)/H_2_O (0.1% TFA) over 30 min). This resulted in 13.5% (1.7 μmol) of unreacted **AuNHC-2**, 5.6% (0.71 μmol) of monopeptide-NHC (**AuNHC-2-pep**), and 6.8% (0.87 μmol) of dipeptide-NHC (**AuNHC-2-pep**_**2**_), which were characterized by RP-HPLC and either HR-DESI-MS or ESI-MS. The purity of both products is >95%.

HR-DESI-MS: Calcd for C_64_H_95_AuN_17_O_10_ (**AuNHC-2-pep**) [M + 2H]^2+^: *m*/*z* = exp 728.8509 (calcd 728.8509).

ESI-MS: Calcd for C_104_H_158_AuN_30_O_16_ (**AuNHC-2-pep**_**2**_)­([M + 3H]^3+^: *m*/*Z* = 761.0 (calcd 762.1882).

### Biological Assays

#### Cell Culture

Murine brain endothelial cells, bEnd.3 (American Type Culture Collection, CRL-2299), were cultured in Dulbecco’s Modified Eagle’s medium (DMEM) supplemented with 10% fetal bovine serum (FBS) (all from Gibco, Thermo Fisher Scientific) in a humidified atmosphere of 5% CO_2_ at 37 °C. The cells were tested for mycoplasma using the LookOut mycoplasma polymerase chain reaction Detection kit (Sigma-Aldrich, Merck). To obtain the in vitro BBB model, 200 μL of a cell suspension with 5000 cells was seeded in the apical side of fibronectin-coated tissue culture polyethylene terephthalate inserts (pore size of 1 μm) for 24-well plates (Corning, Merck), and 500 μL of complete culture medium was added to the basal side of the inserts. The cells were grown for 10 days, and the media of both the apical and basal sides were changed every other day.^46,50^

#### BBB Translocation Assay

bEnd.3 cells grown in the culture inserts for 10 days were incubated with 200 μL of 1 or 5 μM **AuNHC-2-pep** and **AuNHC-2-pep**_**2**_ in the culture medium on the apical side for 30 min or 2 h. The basal side of the inserts was incubated with 500 μL of a regular culture medium. After incubation, samples from both the apical and basal sides were collected and analyzed by ICP–MS for quantification of the Au content. Control inserts cultured with the regular culture medium or with DMSO were also analyzed. For the ICP–MS analysis, the samples were incubated for 24 h with a mixture of HNO_3_ and HCl (1:3) and diluted with ultrapure water to 10 mL. The samples were analyzed in a Thermo ICP–MS X series spectrometer, equipped with an autosampler Cetac ASX-560, with a Mira Mist nebulizer (Burgener), and a conical nebulization chamber, cooled with a Peltier system to 3 °C (plasma power: 1400 W; plasma argon flow: 14 L/min; auxiliary gas flow: 1 L/min; and sample rate: 1 mL/min). Calibration of the equipment was performed with successive dilutions of a standard ICPMS 71 C (INORGANIC VENTURE) in 1:100 solution of HNO_3_/HCl (1:3). One to two replicates were analyzed.

#### BBB Integrity Assay

To evaluate the integrity of the BBB in vitro model after the translocation assay, the fluorescent probe fluorescein isothiocyanate-dextran with a molecular weight of 4 kDa (FD4, Sigma-Aldrich) was used. Initially, the FD4 probe was diluted in transport medium (DMEM FluoroBrite supplemented with 10% FBS, both from Gibco and Thermo Fisher Scientific) to a final concentration of 25 μg/mL. Cells were rinsed twice with phosphate-buffered saline and once with the transport medium. Then, bEnd.3 cells were incubated with 200 μL of the diluted FD4 in the apical side and with 500 μL of regular transport medium in the basal side. After an incubation time of 2 h at 37 °C, 5% CO_2_ samples from both the apical and the basal sides were collected, and their fluorescence was measured (ex. 493 nm/em. 560 nm) in a microplate reader (Varioskan Lux multimode, Thermo Fisher Scientific). Empty inserts were used as controls. Only cell inserts with integrity values above 80% were considered.

#### Viability Assay

The cytotoxicity of the complexes in the bEnd.3 cell line was evaluated using the CellTiter-Glo Cell Viability Assay (Promega). Cells were seeded in 96-well plates at a density of 2 × 10^4^ cells per well in 200 μL of culture medium and cultured overnight for optimal adherence. Stock solutions of the complexes, freshly prepared in DMSO before each experiment, were diluted in the medium with serial dilutions added to the wells before incubation was performed at 37 °C/5% CO_2_ for 24 h. The percentage of DMSO in the cell culture medium did not exceed 2.5%. After 24 h, 100 μL of culture medium was removed from each well and replaced with 40 μL of the CellTiter-Glo Cell Viability Assay. After 15 min of incubation with agitation, 100 μL was transferred to a 96-well white bottom plate, and the luminescence was measured using a microplate reader (Varioskan LUX multimode microplate reader, Thermo Scientific). Each tested condition was determined in at least 4 replicates.

### Radiolabeling

#### Preparation of [^198^Au]­Au­(I)­(THT)­Cl

1.4 mg or 2.8 mg of a solid gold bar was irradiated for up to approximately 10 min at a flux of 1.6 × 10^12^ n/cm^2^ s at the University of Mainz in the research reactor TRIGA to obtain 1 MBq of ^198^Au. [^198^Au]­Au­(0) was dissolved in 40 μL of aqua regia and heated up for 2 min at 90 °C until complete dissolution (Figure 3A). Afterward, the aqua regia was evaporated to dryness. The solid was redissolved in 400 μL of ultrapure water and 200 μL of ethanol to give a yellow solution of AuCl_4_^–^. 10 μL of THT was then added, resulting in a color change of the solution from yellow to colorless, typical of Au­(I) formation. [^198^Au]­Au­(I)­(THT)Cl was used without further purification.

#### Radiolabeling of Au­(I) NHCs

For the manual radiolabeling of the different Au­(I) NHCs, a 2 mL reaction vial was used. 100 μL of the cold Au­(I) NHC precursor (1 mg/100 μL DMSO) was mixed with 100 μL of [^198^Au]­Au­(THT)Cl (approximately 120 KBq, in water/ethanol 66/33) and heated up to 90 °C for 10 min (Figure 3B).

#### Quality Control

For radio-HPLC, 40 μL of the final preparation was diluted with water to 90 μL. 90 μL was injected into the HPLC system via an autosampler.

Analytical HPLC was performed using a Shimadzu gradient system (Kyoto, Japan) equipped with a SPD-20A UV/vis detector. The column for radio-HPLC (RSC Gel C18ec, 125 × 4.0 mm, 5 μm) was purchased from R. Sauerbrey Chromatographie (D-reinhardshagen). Eluents for all HPLC operations were water (solvent A) and acetonitrile (solvent B), both containing 0.1 vol % TFA with a gradient of 0–15 min 0–100% B, 15–20 min 100% B, and 20–22 min 100–0% B. Radioactivity was detected via a HERM LB 500 NaI detector and a Flowstar2 LB514 detector (Berthold Technologies, Bad Wildbad, Germany). A γ-counter (PerkinElmer, Waltham, MA, USA) was used for the analysis of iTLC experiments.

### Stability Studies with HSA

Assessment of ^198^Au NHC compounds’ stability was performed using human serum albumin (H4522-100ML, Sigma-Aldrich), as depicted in Figure 3C. In detail, 100 μL of the respective radiolabeled Au­(I) NHC complex was incubated with 100 μL of human serum albumin at room temperature for 1 h. The protein was precipitated with 100 μL of ice-cold ethanol and 100 μL of ice-cold acetonitrile. After centrifugation, the precipitate (“bound to HSA”) and supernatant (“not bound to HSA”) were analyzed separately with a gamma counter, and the supernatant was additionally analyzed via radio-HPLC. The percentage of stability was calculated from the ratio “bound to HSA” vs “not bound to HSA” and taking into account the radiochemical yield observed of the initial radiolabeling and “not bound to HSA” fraction that was analyzed via radio-HPLC.

### Stability Studies of Nonradioactive Au­(I) NHC Complexes

To replicate the experimental conditions of the radiolabeled complexes, the same conditions were used to prepare [Au­(THT)­Cl], except with no prior irradiation of the gold bar. In detail, solid Au(0) (1 mg) was dissolved in 40 μL of aqua regia and heated to 90 °C for 2 min until complete dissolution. Afterward, the aqua regia was evaporated to dryness, and the solid was redissolved in 400 μL of ultrapure water and 200 μL of EtOH to give a yellow solution of tetrachloroaurate. Thus, 10 μL of THT was added, and the solution changed from yellow to colorless, as previously observed. **AuNHC-2** or **AuNHC-2-pep** (1 mM in DMSO) was then mixed with 100 μL of [Au­(THT)­Cl] solution and heated to 90 °C for 10 min. The samples (40 μL) were then diluted in 90 μL of Milli-Q water and filtered before measuring.

Analytical HPLC measurements were performed using Shimadzu gradient systems (Kyoto, Japan) equipped with an SPD-20A UV–vis detector. The column (MultoKrom 100-5C18, 125 × 4.6 mm) was purchased from CS-Chromatographie Service GmbH (Langerwehe, Germany). Eluents for all HPLC operations were water (solvent A) and acetonitrile (solvent B), both containing 0.1 vol % TFA with a gradient of 0–15 min 0–100% B, 15–20 min 100% B, and 20–22 min 100–0% B. ESI-MS was carried out on an expression^L^ CMS mass spectrometer (Advion Ltd., Harlow, UK) equipped with a quadrupole analyzer and an electron spray ionizer, also in the positive mode.

## Supplementary Material





## Notes

^1^H NMR of AuNHC-2 in DMF-*d*_7_; ^13^C NMR of AuNHC-2 in DMF-*d*_7_; HR-ESI-MS of AuNHC-2; UV chromatogram of AuTMX_2_; UV chromatogram of AuNHC-1; UV chromatogram of AuNHC-2; UV chromatogram of AuNHC-2-pep; HR-DESI-MS of AuNHC-2-pep; ^1^H NMR (500 MHz) of AuNHC-2-pep in DMF-*d*_7_; UV chromatogram of AuNHC-2-pep_2_; ESI-MS of AuNHC-2-pep_2_; ^1^H NMR (500 MHz) of AuNHC-2-pep_2_ in DMF-*d*_7_; ^1^H–^1^H correlation spectroscopy NMR (500 MHz) of AuNHC-2-pep_2_ in DMF-*d*_7_; ^1^H–^1^H nuclear Overhauser enhancement spectroscopy NMR (500 MHz) of AuNHC-2-pep_2_ in DMF-*d*_7_; cytotoxicity studies; and radio-HPLC chromatogram of [^198^Au]­AuNHC-1 in human serum albumin after precipitation of proteins (PDF)
Molecular formula strings (CSV)

## Abbreviations

MeCN: acetonitrile
TBTU: 2-(1*H*-benzotriazole-1-yl)-1,1,3,3-tetramethylaminium tetrafluoroborate
BBB: blood–brain barrier
CO_2_: carbon dioxide
DCM: dichloromethane
DMF: dimethylformamide
DMSO: dimethyl sulfoxide
DIEA: *N*,*N*-diisopropylethylamine
DMEM: Dulbecco’s modified Eagle’s medium
EA: elemental analysis
EtOH: ethanol
FBS: fetal bovine serum
FD: fluorescently labeled dextran
AuNPs: gold nanoparticles
Hz: hertz
HR-DESI-MS: high-resolution desorption electrospray ionization mass spectrometry
HR-ESI-MS: high-resolution electrospray ionization mass spectrometry
HPLC-ESI-MS: high-performance liquid chromatography-electrospray ionization mass spectrometry
HSA: human serum albumin
HCl: hydrochloric acid
HOAt: 1-hydroxy-7-azabenzotriazole
ICP-MS: inductively coupled plasma mass spectrometry
*m*/*z*: mass-to-charge
MeOH: methanol
NHC: *N*-heterocyclic carbenes
HNO_3_: nitric acid
ppm: parts per million
PBS: phosphate buffered saline
PET: polyethylene terephthalate
PCR: polymerase chain reaction
RCP: radiochemical purity
RP-HPLC: reverse-phase HPLC
SPECT: single-photon emission computed tomography
NaOH: sodium hydroxide
SPPS: solid-phase peptide synthesis
THT: tetrahydrothiophene
TLC: thin-layer chromatography
TFA: trifluoroacetic acid
TIPS: triisopropyl silane
TNBC: triple-negative breast cancer
