# Supplementary material for: N‑Heterocyclic Carbenes as Ligands to 198Au(I)-Radiolabeled Compounds: A New Platform for Radiopharmaceutical Design
Source: J Med Chem. 2025 Aug 13;68(16):17516–26. doi: 10.1021/acs.jmedchem.5c01073 (PMC12406186; doi:10.1021/acs.jmedchem.5c01073)
Supplement: Supplementary file 1 [file jm5c01073_si_001.pdf]

Supplementary Information Available

N-Heterocyclic Carbenes as Ligands to ^{198}Au(I) Radiolabeled Compounds: a New Platform for Radiopharmaceutical Design

Sarah Spreckelmeyer,[§,*,a] Sophie R. Thomas,[§,b,c] Franziska Schuderer,[§,c,d] Catarina I. G. Pinto,[e] Ana Luiza de Andrade Querino,[c] Felix A. Böhm,[c] Mihyun Park,[c] Christopher Geppert,[f] Christian Gorges,[f] Filipa Mendes,[e,g] and Angela Casini*[,c]

[a] *Charité - Universitätsmedizin Berlin, corporate member of Freie Universität Berlin, Humboldt-Universität zu Berlin, and Berlin Institute of Health, Department of Nuclear Medicine, Augustenburger Platz 1, 13353, Berlin, Germany.*

[b] *Department of Inorganic Chemistry, University of Vienna, Währinger Straße. 42, 1090 Vienna, Austria.*

[c] *Medicinal and Bioinorganic Chemistry, Department of Chemistry, School of Natural Sciences, Technical University of Munich, Lichtenbergstraße 4, 85748 Garching b. München, Germany.*

[d] *Pharmaceutical Radiochemistry, Department of Chemistry, School of Natural Sciences, Technical University of Munich, Walther-Meißner-Str. 3, 85748 Garching b. München, Germany.*

[e] *C^{2}TN – Centro de Ciências e Tecnologias Nucleares, Instituto Superior Técnico, Universidade de Lisboa, 2695-066 Lisboa, Portugal.*

[f] *Forschungsreaktor TRIGA Mainz, Johannes Gutenberg-Universität Mainz, Fritz-Strassmann-Weg 2, 55128 Mainz, Germany.*

[g] *DECN – Departamento de Engenharia e Ciências Nucleares, Instituto Superior Técnico, Universidade de Lisboa, 2695-066 Lisboa, Portugal.*

§ Co-first authors.

*Corresponding authors:

sarah.spreckelmeyer@charite.de; angela.casini@tum.de

SI Content:

Figure S1 – ^{1}H NMR of **AuNHC-2** in DMF-d_7.

Figure S2 – ^{13}C NMR of **AuNHC-2** in DMF-d_7.

Figure S3 – HR-ESI-MS of **AuNHC-2**: the inset shows the isotopic pattern of the species [M+] (m/Z 633.1782).

Figure S4 - UV-chromatogram of **AuTMX$_2$**.

Figure S5 - UV-chromatogram of **AuNHC-1**.

Figure S6 - UV-chromatogram of **AuNHC-2**.

Figure S7 - UV-chromatogram of **AuNHC-2-pep**.

Figure S8 – A: HR-DESI-MS of **AuNHC-2-pep**: the inset shows the isotopic pattern of the species $[M+2H]^{2+}$ (m/Z 728.8509, mass error = 0.0 ppm); B: HR-ESI-MS of **AuNHC-2-pep**: the inset shows the isotopic pattern of the species $[M+H]^{2+}$.

Figure S9 – ^{1}H NMR (500 MHz) of **AuNHC-2-pep** in DMF-d_7.

Figure S10 - UV-chromatogram of **AuNHC-2-pep$_2$**.

Figure S11 – A: HR-DESI-MS of **AuNHC-2-pep$_2$**; B: HR-ESI-MS of **AuNHC-2-pep$_2$**. The inset shows the isotopic pattern of the species $[M+3H]^{4+}$.

Figure S12 – ^{1}H NMR (500 MHz) of **AuNHC-2-pep$_2$** in DMF-d_7.

Figure S13 – ^{1}H-^{1}H COSY NMR (500 MHz) of **AuNHC-2-pep$_2$** in DMF-d_7.

Figure S14 – ^{1}H-^{1}H NOESY NMR (500 MHz) of **AuNHC-2-pep$_2$** in DMF-d_7.

Figure S15 – Cytotoxicity studies.

Figure S16 – Radio-HPLC chromatogram of [^{198}Au]**AuNHC-1** in human serum albumin after precipitation of proteins.

Original source data are available on Zenodo repository with DOI: 10.5281/zenodo.16539101

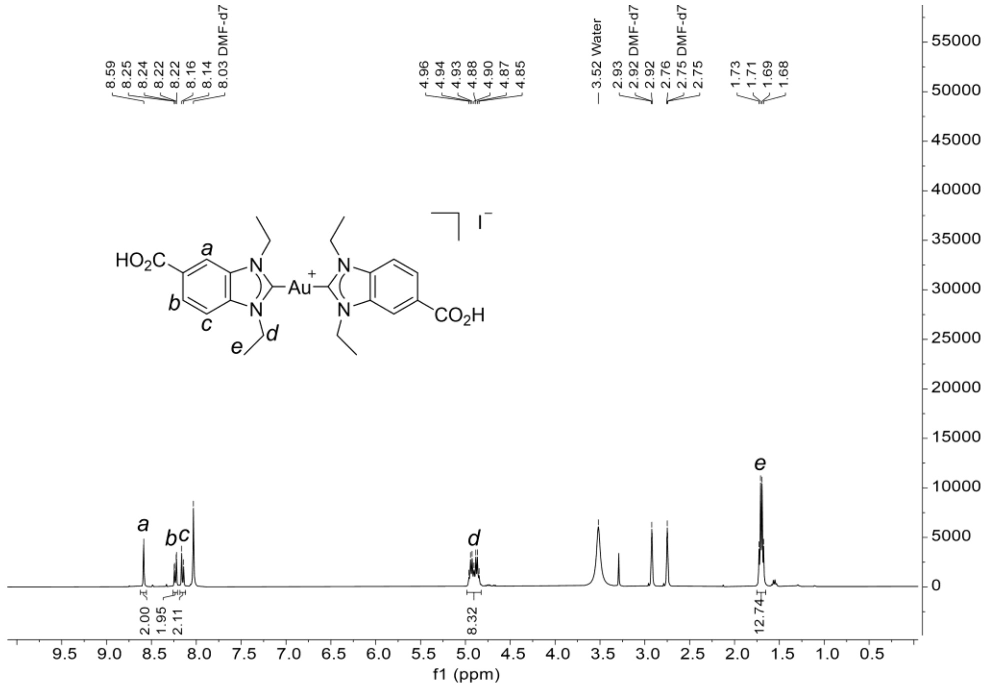

Figure S1 – ^{1}H NMR of **AuNHC-2** in DMF-*d*$_7$.

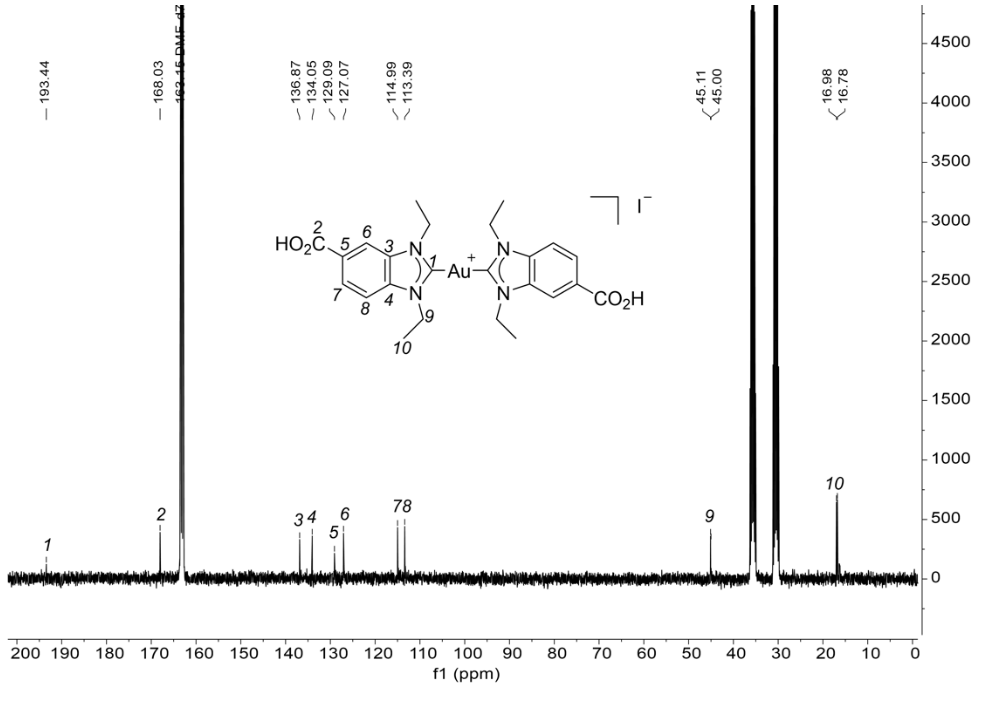

Figure S2 – ^{13}C NMR of **AuNHC-2** in DMF-*d*$_7$.

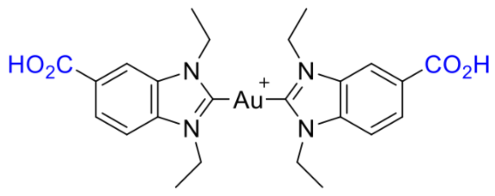

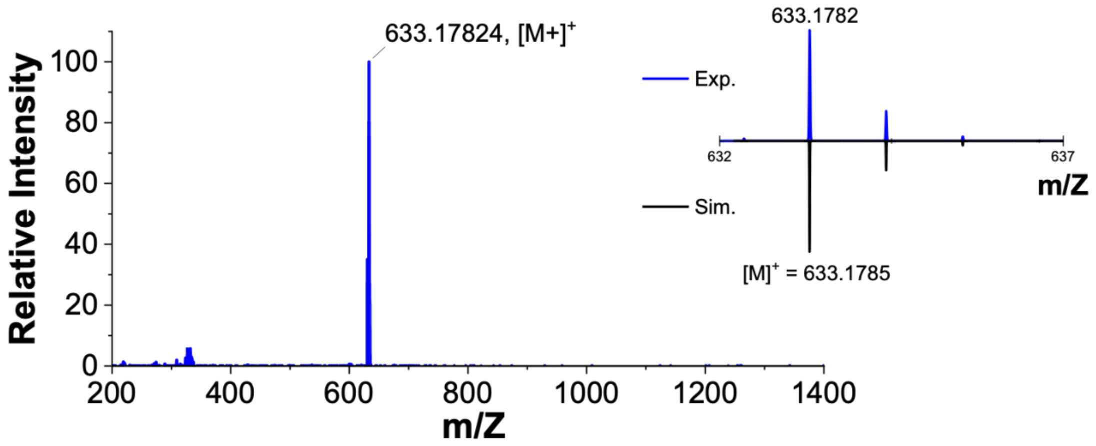

Figure S3 – HR-ESI-MS of **AuNHC-2**: the inset shows the isotopic pattern of the species [M+] (m/Z 633.1782).

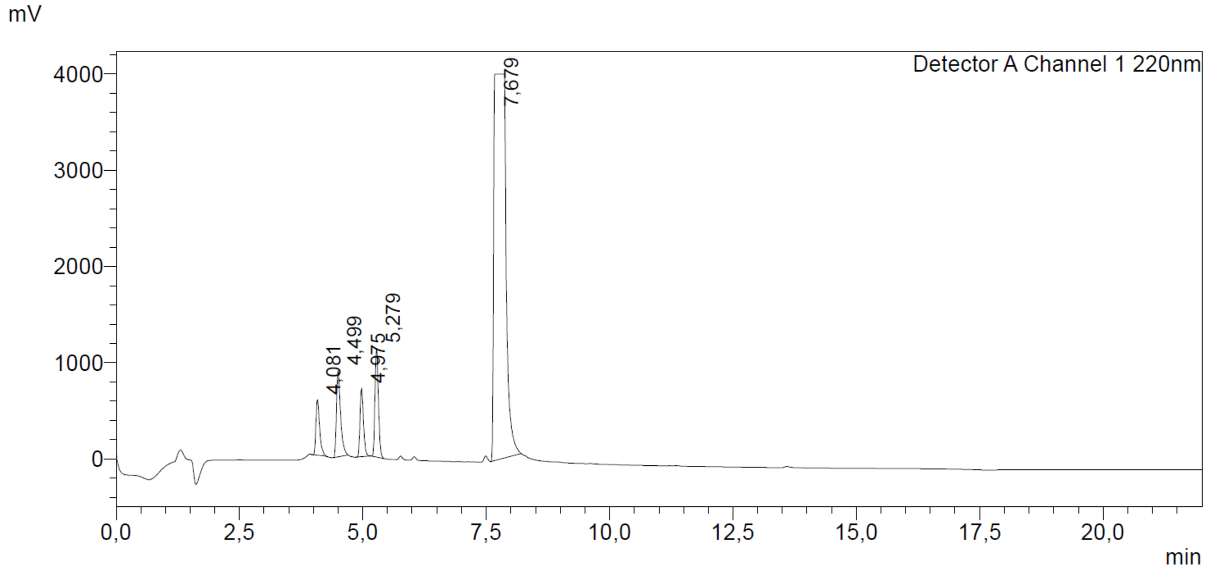

Figure S4 – UV-chromatogram of **AuTMX2** (t_R = 7.679 min).

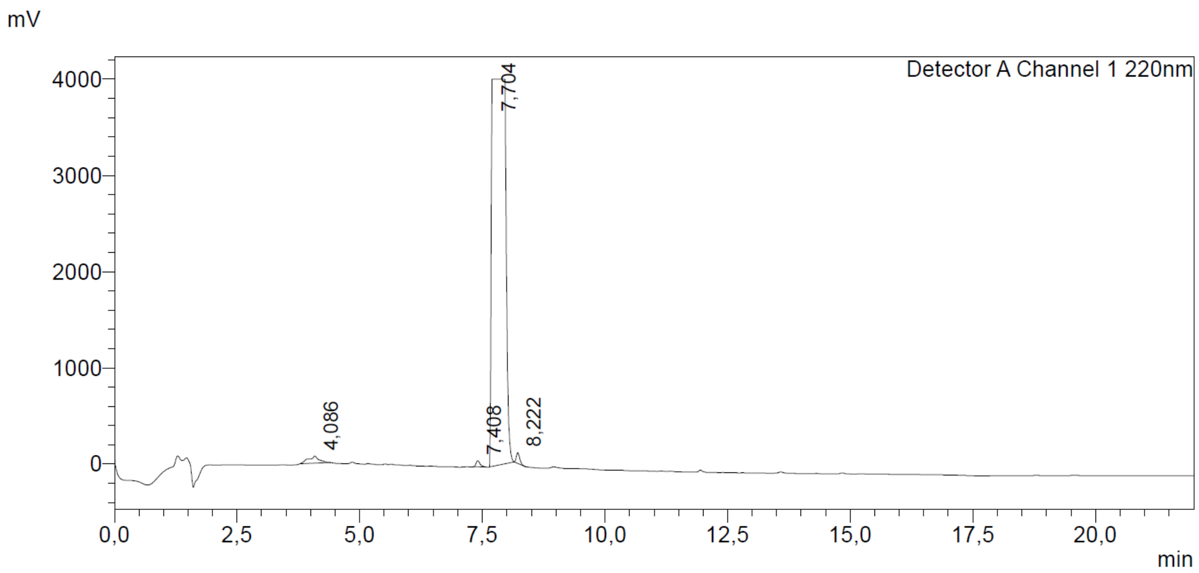

Figure S5 – UV-chromatogram of **AuNHC-1** (t_R = 7.70 min).

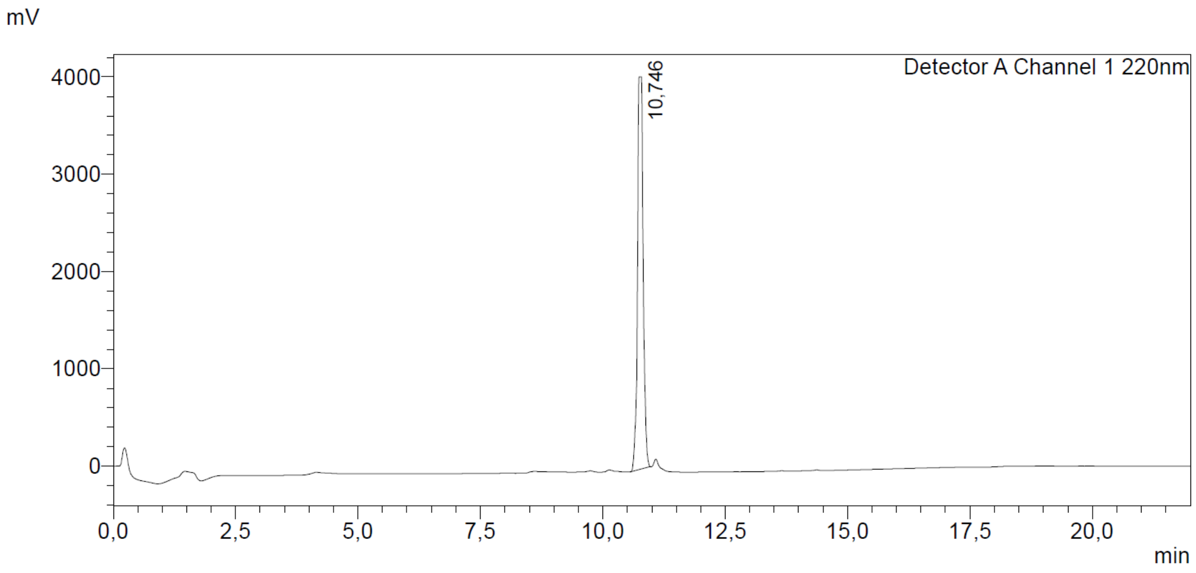

Figure S6 – UV-chromatogram of **AuNHC-2** (t_R = 10.75 min).

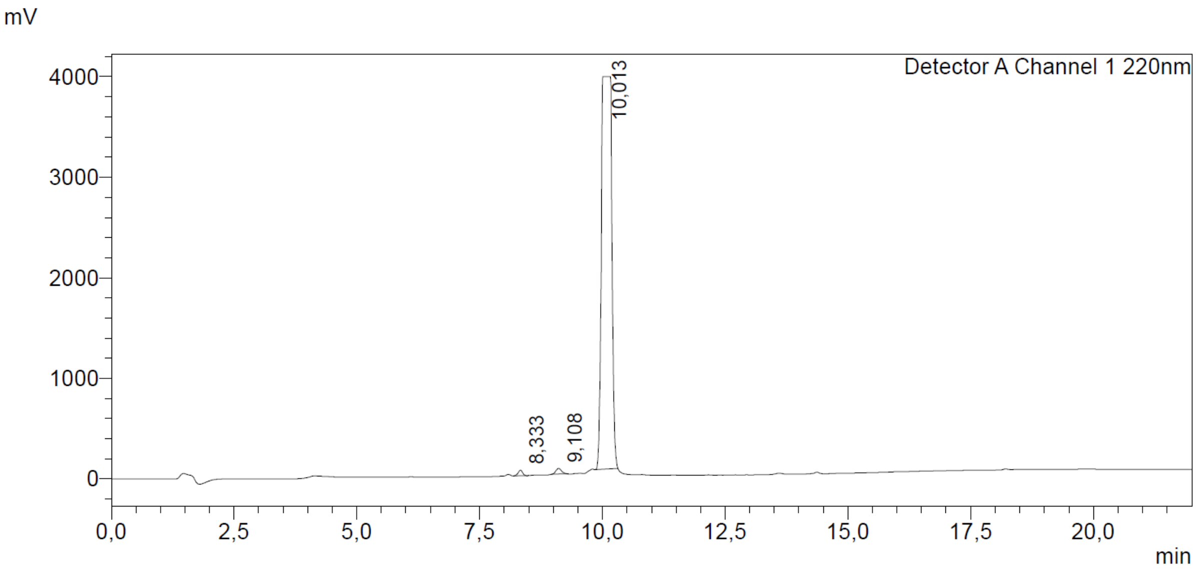

Figure S7 – UV-chromatogram of **AuNHC-2-pep** (t_R = 10.01 min).

A)

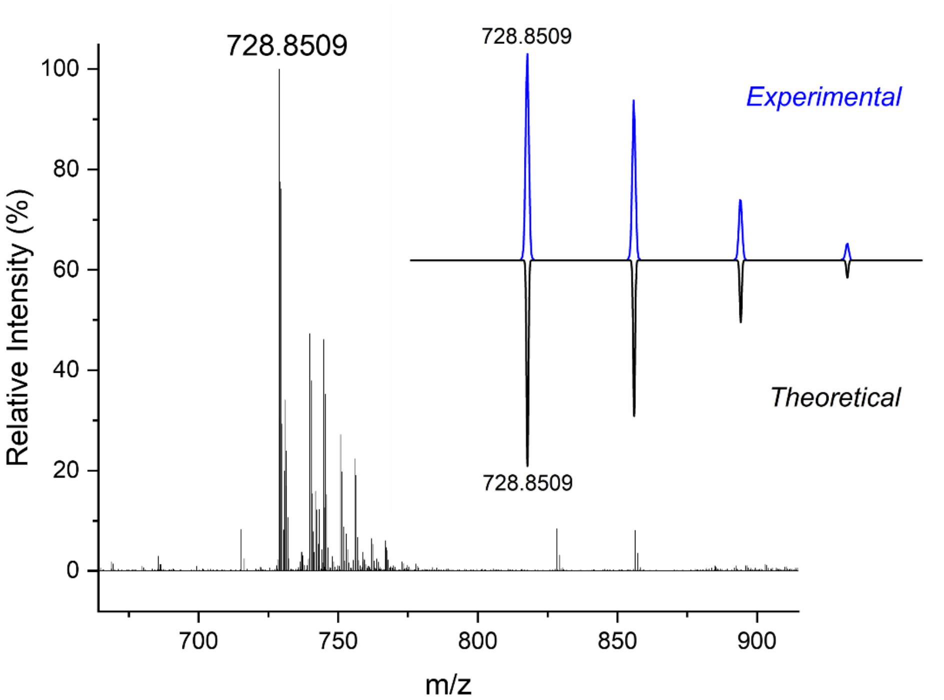

B)

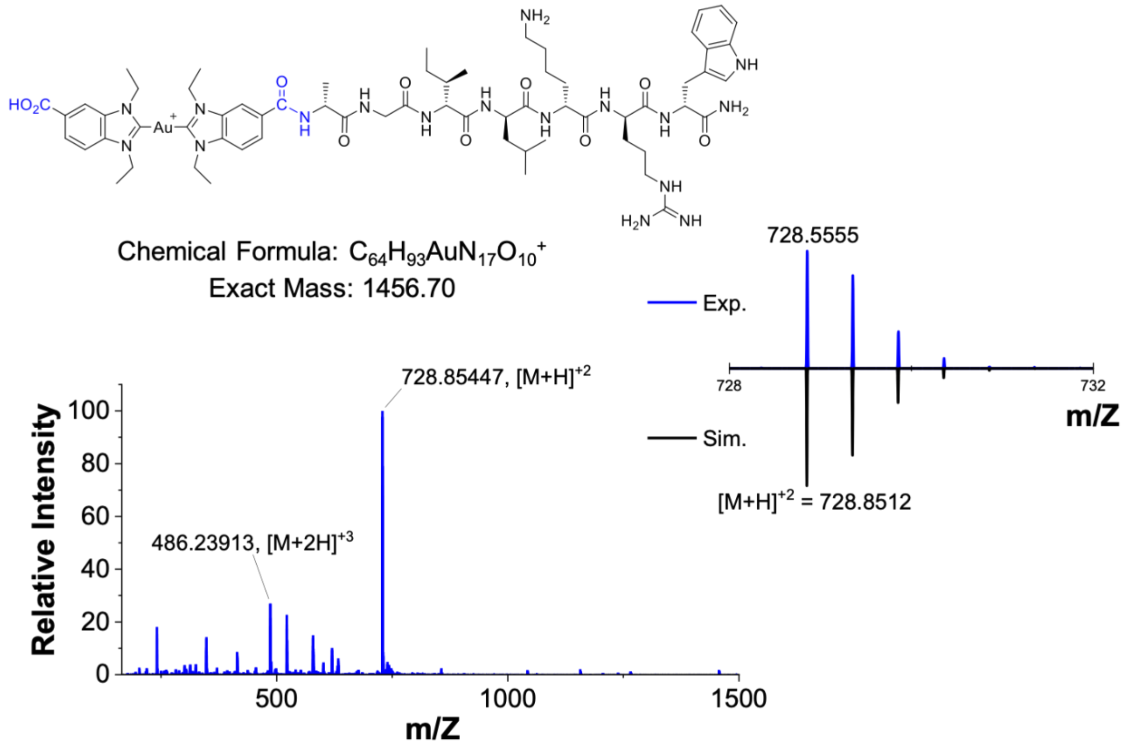

Figure S8 – A: HR-DESI-MS of **AuNHC-2-pep**: the inset shows the isotopic pattern of the species $[M+2H]^{2+}$ (m/Z 728.8509, mass error = 0.0 ppm). B: HR-ESI-MS of **AuNHC-2-pep**: the inset shows the isotopic pattern of the species $[M+H]^{2+}$.

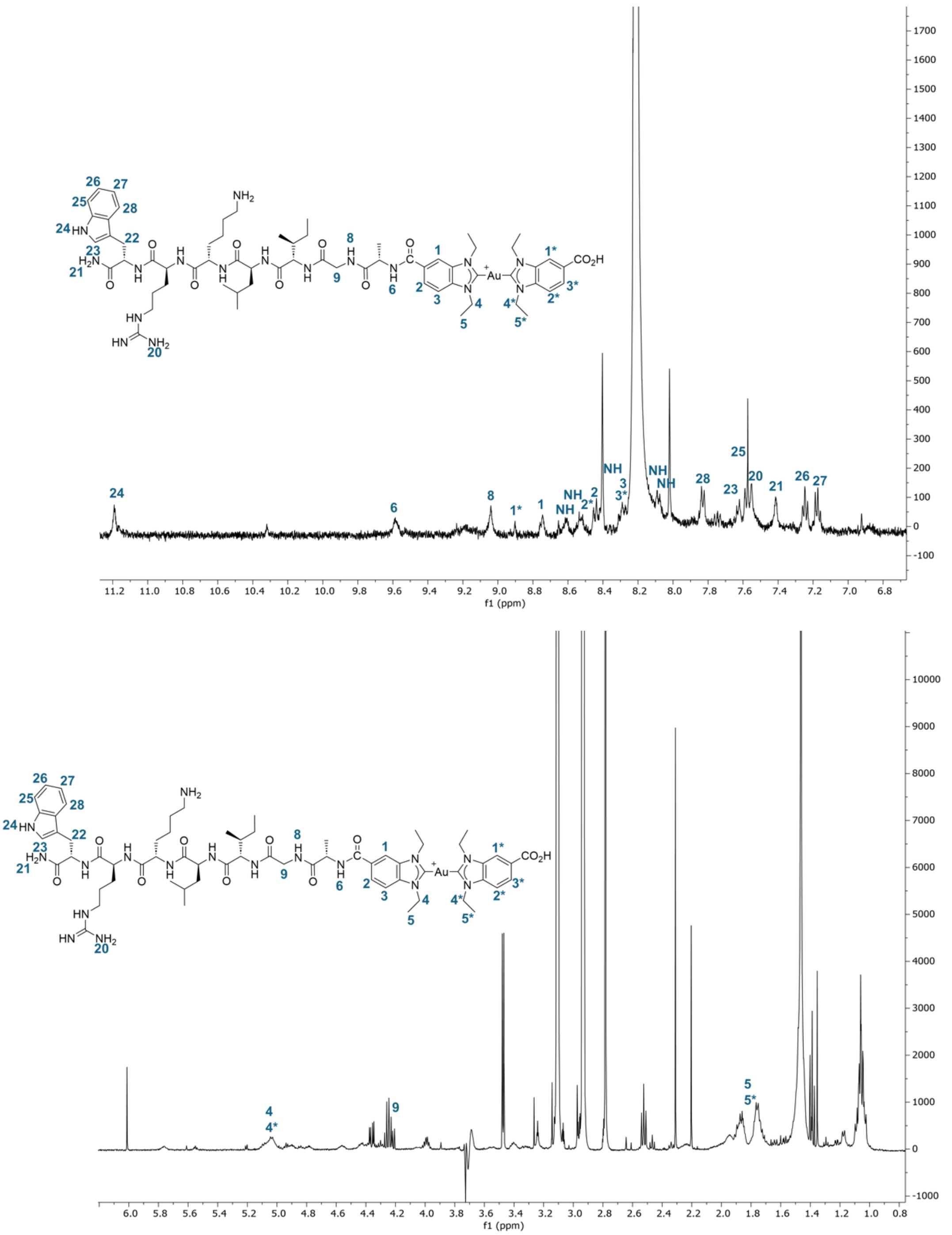

Figure S9 – ^{1}H NMR (500 MHz) of **AuNHC-2-pep** in DMF-d_7.

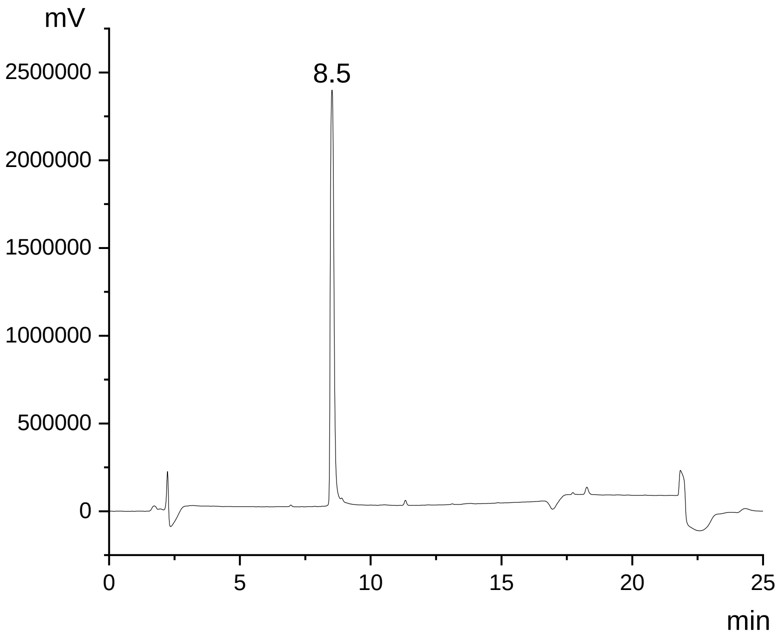

Figure S10 – UV-chromatogram of **AuNHC-2-pep$_2$** (t_R = 8.5 min).

A)

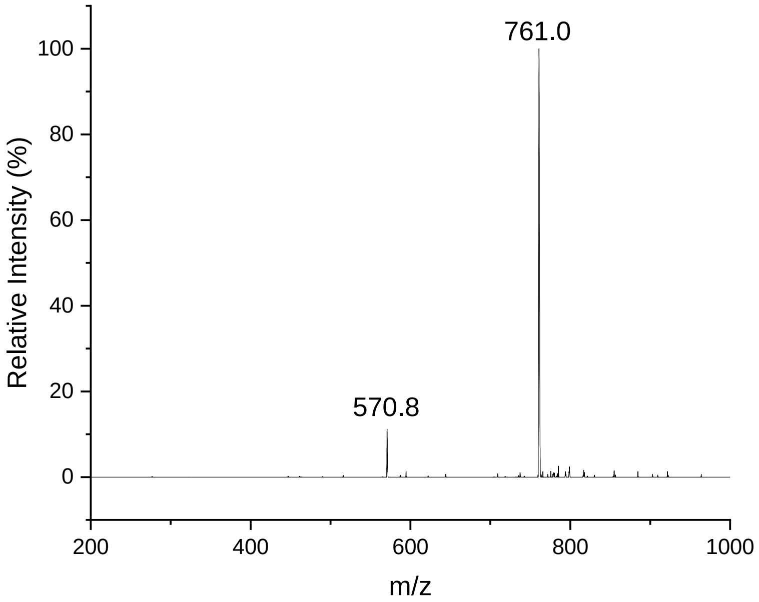

B)

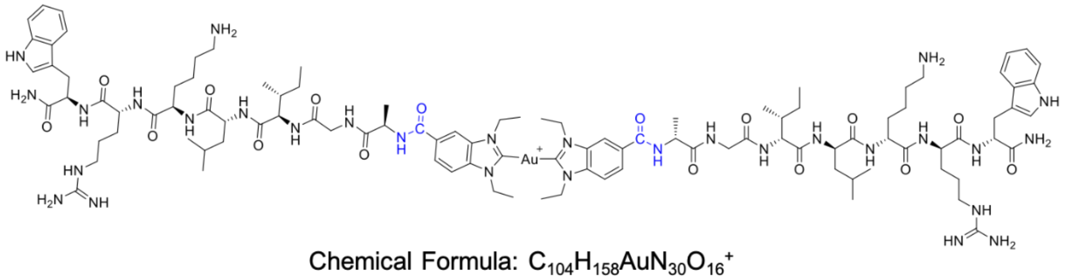

Chemical Formula: $C_{104}H_{158}AuN_{30}O_{16}^{+}$
Exact Mass: 2280.21

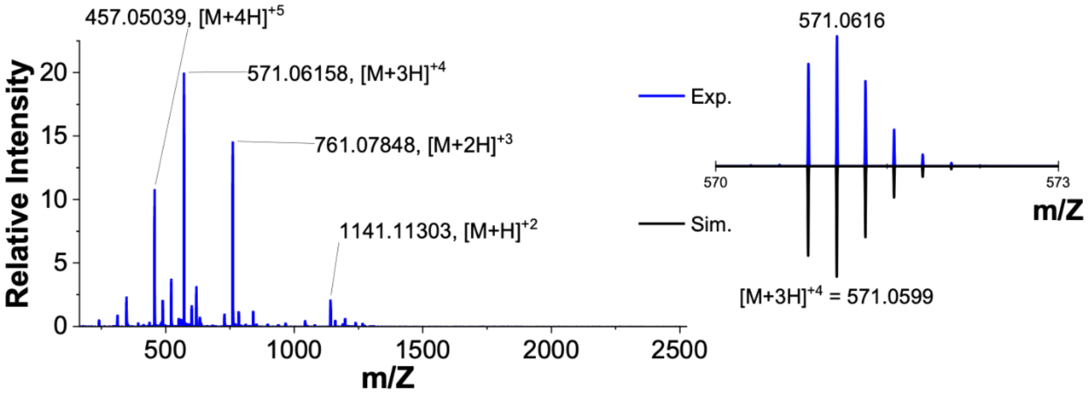

Figure S11 – A: ESI-MS of **AuNHC-2-pep$_2$**; B: HR-ESI-MS of **AuNHC-2-pep$_2$.** The inset shows the isotopic pattern of the species $[M+3H]^{4+}$.

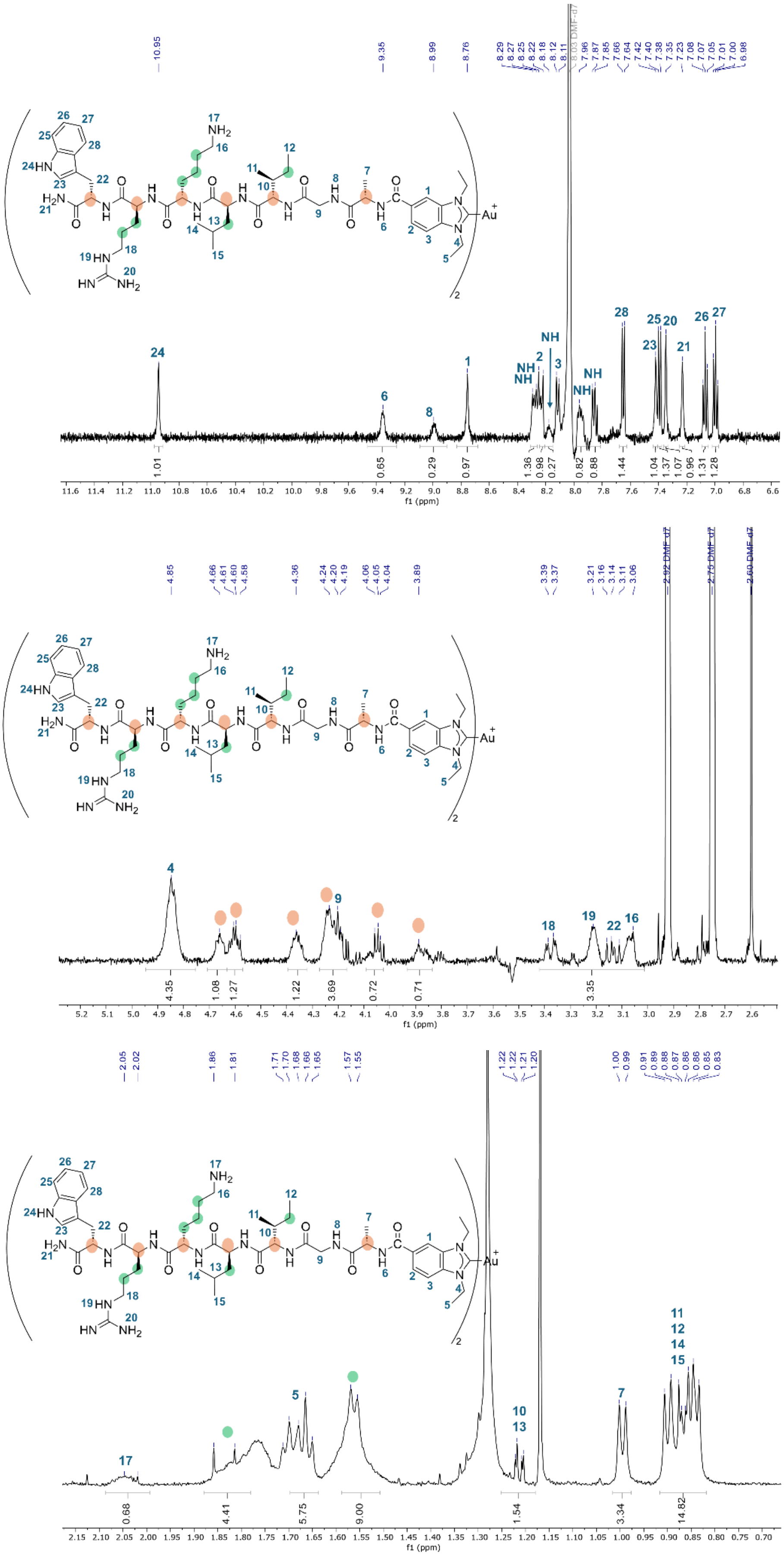

Figure S12 – ^{1}H NMR (500 MHz) of **AuNHC-2-pep$_2$** in DMF-d_7.

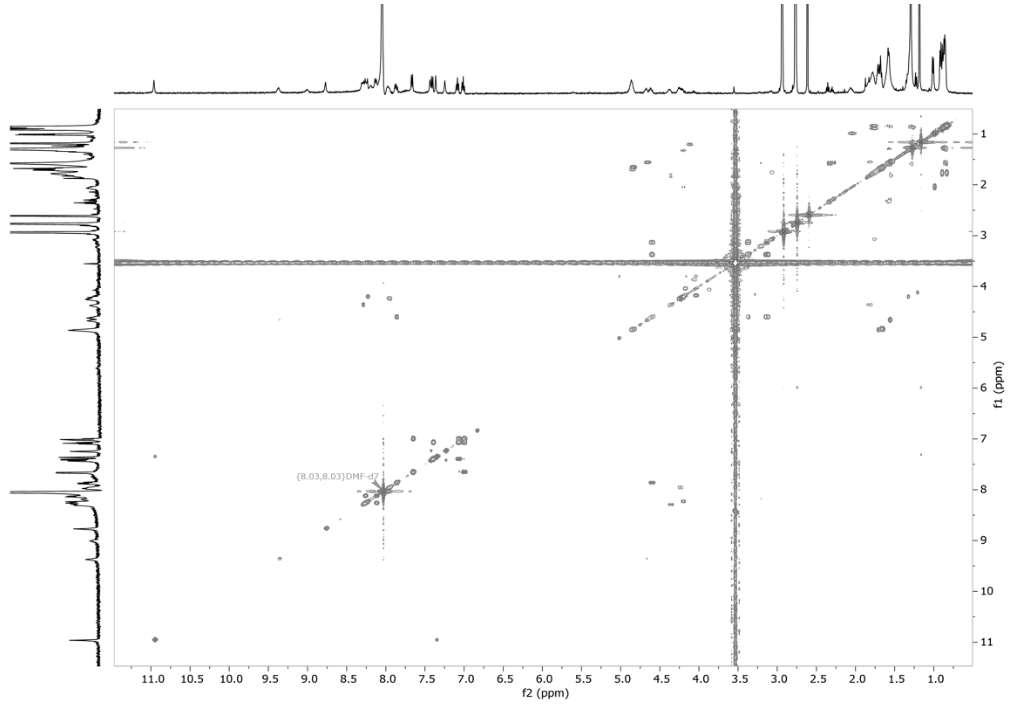

Figure S13 – ^{1}H-^{1}H COSY NMR (500 MHz) of **AuNHC-2-pep$_2$** in DMF-d_7.

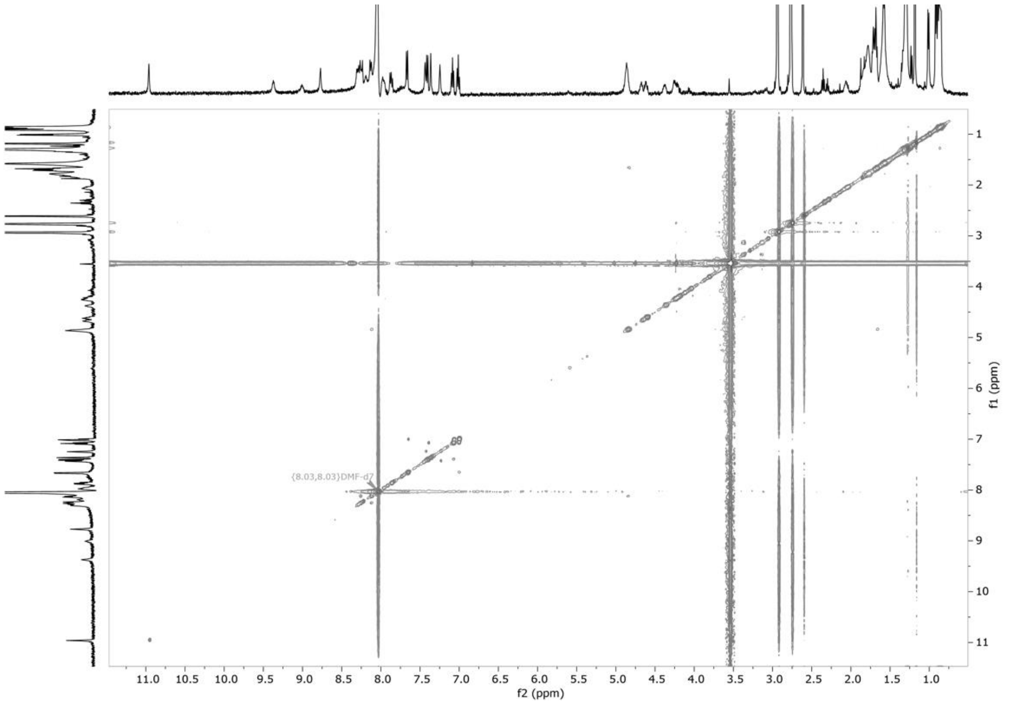

Figure S14 – ^{1}H-^{1}H NOESY NMR (500 MHz) of **AuNHC-2-pep$_2$** in DMF-d_7.

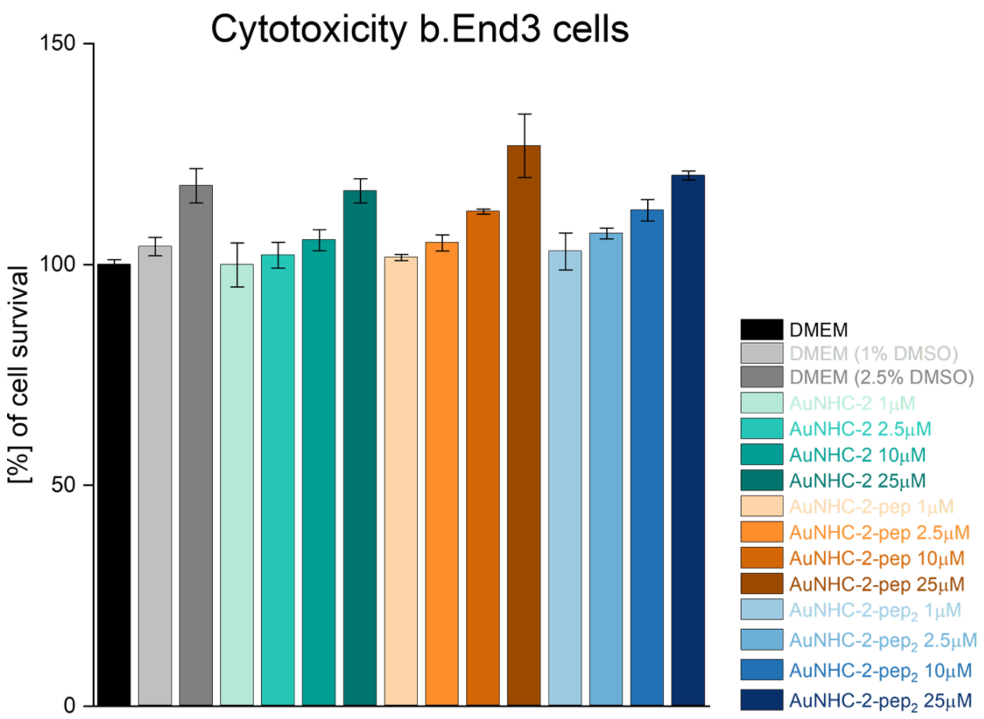

Figure S15 – Cytotoxicity study of **AuNHC-2**, **AuNHC-2-pep**, and **AuNHC-2-pep2** on b.End3 cells assessed with CellTiter-Glo. DMSO content of 10 uM and 25 uM samples was 1% and 2.5%, respectively. Neither the DMSO content nor concentrations of up to 25 uM of Au(I) complexes showed a cytotoxic effect.

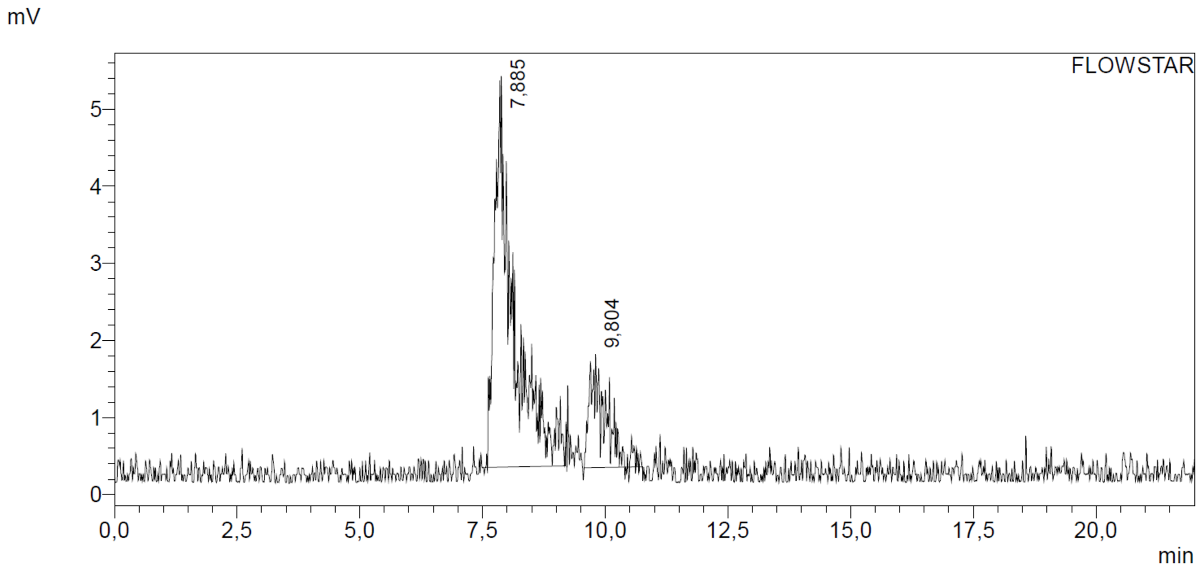

Figure S16 – Radio-HPLC chromatogram of [^{198}Au]**AuNHC-1** in human serum albumin after precipitation of proteins. Area of peak at t_R = 7.9 min = 79.7%.
